# Reconstruction of cell population dynamics using CFSE

**DOI:** 10.1186/1471-2105-8-196

**Published:** 2007-06-12

**Authors:** Andrew Yates, Cliburn Chan, Jessica Strid, Simon Moon, Robin Callard, Andrew JT George, Jaroslav Stark

**Affiliations:** 1 Department of Biology, Emory University, 1510 Clifton Road, Atlanta, GA 30322, USA; 2 Department of Biostatistics and Bioinformatics, Duke University Laboratory of Computational Immunology, 106 North Bldg, Research Drive, Box 90090, Durham, NC 27708, USA; 3 Peter Gorer Department of Immunobiology, Guy's, King's and St Thomas' School of Medicine, King's College London, Guy's Hospital, London SE1 9RT, UK; 4 Department of Mathematics, Imperial College London, 180 Queen's Gate, London SW7 2BZ, UK; 5 Centre for Integrative Systems Biology at Imperial College (CISBIC), UK; 6 Immunobiology Unit, Institute of Child Health, 30 Guilford Street, London WC1N 1EH, UK; 7 Department of Immunology, Faculty of Medicine, Imperial College London, Hammersmith Hospital, London W12 0NN, UK

## Abstract

**Background:**

Quantifying cell division and death is central to many studies in the biological sciences. The fluorescent dye CFSE allows the tracking of cell division *in vitro *and *in vivo *and provides a rich source of information with which to test models of cell kinetics. Cell division and death have a stochastic component at the single-cell level, and the probabilities of these occurring in any given time interval may also undergo systematic variation at a population level. This gives rise to heterogeneity in proliferating cell populations. Branching processes provide a natural means of describing this behaviour.

**Results:**

We present a likelihood-based method for estimating the parameters of branching process models of cell kinetics using CFSE-labeling experiments, and demonstrate its validity using synthetic and experimental datasets. Performing inference and model comparison with real CFSE data presents some statistical problems and we suggest methods of dealing with them.

**Conclusion:**

The approach we describe here can be used to recover the (potentially variable) division and death rates of any cell population for which division tracking information is available.

## Background

Quantifying the dynamics of cell populations involves measuring rates of division and death. On a practical level, knowledge of these rates can be important for the clinical assessment of diseases characterised by dysregulated cell populations such as neoplasias. Perhaps more fundamentally, quantifying cell dynamics is important for testing hypotheses regarding the population biology of cells.

Studies of cell proliferation have benefited in recent years from the development of a method to measure the number of divisions single cells have undergone using CFSE (Carboxy Fluoroscein Succinimidyl Ester), a fluorescent and cell-membrane impermeable dye. CFSE is now used widely in immunology to study lymphocyte dynamics [1] but also in oncology [2], stem cell research [3,4] and to study the kinetics of bacterial division [5]. Briefly, the procedure is as follows. A population of cells is stained with CFSE, and the dye contained in each cell is shared approximately equally among daughter cells upon division. The fluorescence intensities of the population of CFSE-labeled cells can then be measured at a later time using flow cytometry. Cohorts of cells that have undergone the same number of divisions are usually observed to have approximately log-normally distributed intensities, with median decreasing roughly two-fold with each division. Analysis of CFSE profiles allows the estimation of the proportions of cells in culture that are in each generation. These proportions can indicate the extent of division in a population, but CFSE information can also be used to simultaneously quantify division and death if the total numbers of live cells in each generation are known at two or more timepoints. In *in vitro *experiments, these can be estimated by adding known numbers of fluorescent beads to the culture, sampling from it, counting both cells and beads in the sample using flow cytometry and scaling the generation proportions appropriately.

The information CFSE provides regarding this generational structure augments methods of pulse-labelling with markers such as BrDU (5-bromo-2'-deoxyuridine) or tritiated thymidine, which have traditionally been used to quantify proliferation. These compounds are taken up during DNA synthesis and allow the measurement of the proportion of the population undergoing mitosis during the labelling period. This technique has been used in conjuction with mathematical models to quantify the turnover of populations that are essentially homogeneous (see, for example, [6]). Models have been used to quantify turnover from CFSE data in similar situations [7-11]. In these studies, all cells are considered to be identical, and death or entry into division are represented as Poisson processes. ODEs are usually used, providing the expected numbers of cells in each division. While these models are useful as a starting point, in their simplest form they allow for arbitrarily short inter-division times. This is a biologically unrealistic artifact which can lead to difficulties in the interpretation of estimates of average division and death rates [12]. Other CFSE modeling studies have overcome this by turning to the classic Smith-Martin model of the cell cycle [13]. In this model cells are assumed to spend exponentially-distributed times in a quiescent A-phase before progressing deterministically through an 'actively dividing' B-phase (roughly corresponding to DNA synthesis and mitosis) of finite duration. However, if different susceptibilities to death are allowed in the two phases, as might reasonably be expected given the metabolic differences between quiescence and mitosis, it has been shown that CFSE data alone is not sufficient to identify all parameters of the general Smith-Martin model [9,10], and additional information (such as the proportion of cells in each generation that are in the A- and B- phases) is required.

As a further complication, it has increasingly been recognised that rates of division and death are usually not homogeneous, and that it is essential to consider this if CFSE is to be used as a practical tool for studying cell dynamics in any depth. Rates of division and death typically vary systematically at a population level. This variation might occur with the number of divisions a cell has undergone; with time, for example as the availability of nutrients, inter-cellular signalling molecules or pro- or anti-apoptotic factors changes over the course of an experiment; or both. Some of these issues were tackled in a series of elegant studies by Gett and Hodgkin [14], Deenick et al. [15], and the subsequent extension of their analysis by de Boer and colleagues [12,16]. They quantified the kinetics of *in vitro *stimulation of CFSE-labeled T cells, using a hybrid model in which entry into the first division is stochastic and subsequent divisions are deterministic. They discuss the estimation of the distribution of entry times into the first division, and showed a significant improvement in fit using a division-dependent death rate. Towards a more general approach, Leon *et al*. [17] proposed a framework for modeling asynchronous division with CFSE data and used this to determine the parameters of probability distributions of inter-division times, allowing for heterogeneity in cell kinetics with respect to division history. However, their analytic approach and the lack of treatment of the sources of discrepancy between model and data make the fitting and comparison of models difficult, and so limits its practical usefulness.

**Figure 1:**
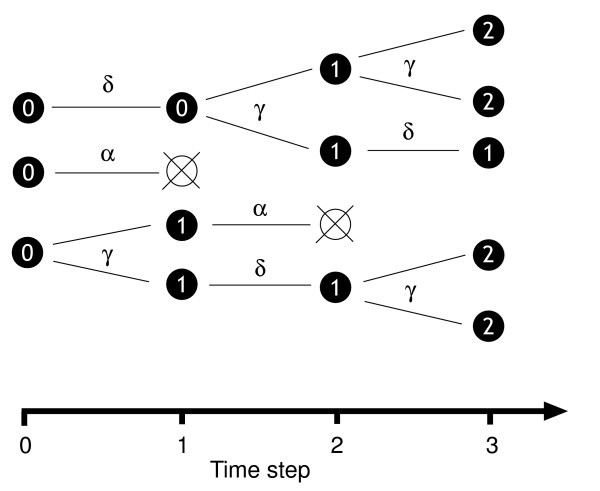
**A simple branching process in discrete time**. A schematic representation of a branching process. The numbers in the circles denote the generation of the cell or the number of divisions it has undergone since being labeled with CFSE. We begin with a population of undivided cells at time 0. In each timestep, each cell divides with probability *γ*, survives without dividing with probability *δ *and dies with probability *α *= 1 - *γ *- *δ*. At a later timestep *t*, sorting cells according to their CFSE content allows the numbers of cells in each generation to be estimated. The formalism we describe in this paper allows us to calculate the moments of the probability distribution of these counts at one timestep given knowledge of the number of cells in each generation at an earlier time.

In this paper we present a distinct and complementary method of modeling CFSE data. We use discrete-time branching processes to describe heterogeneous cell kinetics and suggest a likelihood-based method of inference. Branching processes have been applied successfully to model cell growth in many areas in biology [18-22]. In such models, cells are considered to act independently and divide and die according to probabilistic rules. In a discrete-time process a cell is assumed to either divide once, die or survive undivided in each discrete time interval (Figure 1).

The method we present here has at least two advantages over existing approaches. Firstly, in many cases even time-series of CFSE data may be insufficient to identify the parameters of more detailed models of cell division, and in some cases (as in the general Smith-Martin model discussed above) unique identification of all parameters with CFSE alone is not possible. In contrast, branching processes make minimal assumptions regarding the cell cycle – essentially, the finite timestep imposes a lower bound on the time required to complete a division – and in general all of their parameters are identifiable. In particular this allows useful dynamical information to be recovered even from limited CFSE datasets, such as a single timepoint. Secondly, the inference procedure we propose provides a statistically sound basis for model fitting. Many studies (implicitly) ascribe the discrepancies between the model and the counts of cells in each generation recovered from CFSE profiles as measurement error terms of constant variance. In this paper we challenge this assumption and use a standard stochastic description of cell population dynamics, along with a more realistic treatment of the sources of discrepancy between model and data, to provide the appropriate weighting to each observation when fitting models. Specifically, when estimating parameters of stochastic models from data it is important to assess the relative contributions of fluctuations arising from the intrinsically probabilistic nature of cell dynamics and measurement error or other forms of experimental noise. In this paper we describe two frameworks for parameter estimation; one when fluctuations are the most important form of discrepancy between model and data, and the other when other forms of measurement error dominate. In the latter case, the procedure we describe in this paper can be applied to any model used to describe CFSE data that provides the expected cell counts in each generation.

Using a likelihood-based estimation method requires calculating the probability (likelihood) of a set of observations arising given a model. The generating-function approach we describe allows us in principle to write an exact likelihood given a specification of a branching process model, initial cell numbers, and experimentally observed cell counts at one or more timepoints. However, this method becomes impractical when used with more than a few cells or one or two cell divisions, and is essentially impossible to apply to experimental situations which involve typically tens of thousands of cells. We propose a solution to this problem with the use of a Quasi-Likelihood estimation method. This requires only the first two moments of the probability distribution of the total numbers of cells in each generation – that is, their expectation values and their variance-covariance matrix. We will show that this key simplification allows the model parameters to be inferred from CFSE information.

## Results

In Section 1 we describe the theory underlying the parameter estimation and in Section 2 we validate it using synthetic datasets. In Section 3 we describe how to deal with statistical issues that may arise with the application of the method to experimental data, and illustrate this with an analysis of data from an *in vitro *T cell proliferation experiment.

### 1. Cell kinetics as a branching process

#### Calculating the probability distribution of cell counts

To apply a maximum likelihood method to estimate parameters of a stochastic model of cell division and death from CFSE data, we need to characterise the probability distribution of cell counts predicted by the model. In this section we outline this calculation for a general branching process model in discrete time, or a Galton-Watson process [23].

In these models, during each timestep a cell can do one of the following: divide, with probability *γ*; survive without dividing, with probability *δ*; or die, with probability 1 - *γ *- *δ *(Figure 1). A particular model of the kinetics of a cell population specifies these probabilities, which in the simplest case might be assumed to be constant. In general they may depend on either the number of divisions the cell has undergone (which we refer to as the generation number), explicitly on time, or both. The key assumptions are that all cells act independently, their offspring generate their own branching processes according to the same rules, and that cells retain no memory of events in previous timesteps other than the total number of divisions they have undergone.

The parameters of biological interest are usually *γ *and *α *(the probabilities of division and death). However, in the formalism we use here it proves simpler to work with the quantities *γ *and *δ *(the probability of survival without division). The probability of death *α *can then be calculated from 1 - *γ *- *δ*. A particular branching process model of cell division is specified by a choice of timestep, a starting condition – the number of cells in each generation at a given time, usually all in generation 0 – and a set of parameters that determine the probabilities *γ*_*i *_(*t*) and *δ*_*i *_(*t*) for each generation at each subsequent timestep.

Let the state of the cell population at timestep *t *be the vector Zt=(Zt0,Zt1,...Ztn)
 MathType@MTEF@5@5@+=feaafiart1ev1aaatCvAUfKttLearuWrP9MDH5MBPbIqV92AaeXatLxBI9gBaebbnrfifHhDYfgasaacH8akY=wiFfYdH8Gipec8Eeeu0xXdbba9frFj0=OqFfea0dXdd9vqai=hGuQ8kuc9pgc9s8qqaq=dirpe0xb9q8qiLsFr0=vr0=vr0dc8meaabaqaciaacaGaaeqabaqabeGadaaakeaaieqacqWFAbGwdaWgaaWcbaGaemiDaqhabeaakiabg2da9maabmaabaGaemOwaO1aa0baaSqaaiabdsha0bqaaiabicdaWaaakiabcYcaSiabdQfaAnaaDaaaleaacqWG0baDaeaacqaIXaqmaaGccqGGSaalcqGGUaGlcqGGUaGlcqGGUaGlcqWGAbGwdaqhaaWcbaGaemiDaqhabaGaemOBa4gaaaGccaGLOaGaayzkaaaaaa@4283@, where the components Zti
 MathType@MTEF@5@5@+=feaafiart1ev1aaatCvAUfKttLearuWrP9MDH5MBPbIqV92AaeXatLxBI9gBaebbnrfifHhDYfgasaacH8akY=wiFfYdH8Gipec8Eeeu0xXdbba9frFj0=OqFfea0dXdd9vqai=hGuQ8kuc9pgc9s8qqaq=dirpe0xb9q8qiLsFr0=vr0=vr0dc8meaabaqaciaacaGaaeqabaqabeGadaaakeaacqWGAbGwdaqhaaWcbaGaemiDaqhabaGaemyAaKgaaaaa@30E2@ are random variables that represent the number of live cells that have divided *i *times. The maximum division number *n *is chosen to be at the limit of detectability on a CFSE profile, or the maximum division number of interest. Given a model and a dataset consisting of the cell counts in each generation at two or more timepoints, we wish to estimate the model parameters. To do this we use the data and the joint probability distribution of **Z**_*t *_at each timepoint to construct a likelihood. Maximising this with respect to the model parameters and the timestep provides us with best-fit estimates.

We use a probability-generating function (pgf) approach, described in detail in Methods, which allows us to calculate the moments of the distribution of cell numbers in each generation at one timestep given knowledge of their numbers in the previous timestep. Derivatives of the pgf are used to construct a transition matrix **M **which maps a measured set of cell counts **Z**_*t *_to their expected values *E *(**Z**_*t*+1_) at the following timestep. For stationary (time-independent) parameters, we show in the Methods section that given any set of initial cell counts Z0=(Z00,Z01,...Z0n)
 MathType@MTEF@5@5@+=feaafiart1ev1aaatCvAUfKttLearuWrP9MDH5MBPbIqV92AaeXatLxBI9gBaebbnrfifHhDYfgasaacH8akY=wiFfYdH8Gipec8Eeeu0xXdbba9frFj0=OqFfea0dXdd9vqai=hGuQ8kuc9pgc9s8qqaq=dirpe0xb9q8qiLsFr0=vr0=vr0dc8meaabaqaciaacaGaaeqabaqabeGadaaakeaaieqacqWFAbGwdaWgaaWcbaGaeGimaadabeaakiabg2da9maabmaabaGaemOwaO1aa0baaSqaaiabicdaWaqaaiabicdaWaaakiabcYcaSiabdQfaAnaaDaaaleaacqaIWaamaeaacqaIXaqmaaGccqGGSaalcqGGUaGlcqGGUaGlcqGGUaGlcqWGAbGwdaqhaaWcbaGaeGimaadabaGaemOBa4gaaaGccaGLOaGaayzkaaaaaa@4077@

*E *(**Z**_*t*_|**Z**_0_) = **Z**_**0**_**M**^*t*^,

where

M=(δ02γ00⋯⋯⋯0δ12γ10⋯⋯00δ22γ20⋯⋮⋮⋮⋮⋱⋮⋯⋯⋯0δn−12γn−1⋯⋯⋯00δn)
 MathType@MTEF@5@5@+=feaafiart1ev1aaatCvAUfKttLearuWrP9MDH5MBPbIqV92AaeXatLxBI9gBaebbnrfifHhDYfgasaacH8akY=wiFfYdH8Gipec8Eeeu0xXdbba9frFj0=OqFfea0dXdd9vqai=hGuQ8kuc9pgc9s8qqaq=dirpe0xb9q8qiLsFr0=vr0=vr0dc8meaabaqaciaacaGaaeqabaqabeGadaaakeaaieqacqWFnbqtcqGH9aqpdaqadaqaauaabeqagyaaaaaabaacciGae4hTdq2aaSbaaSqaaiabicdaWaqabaaakeaacqaIYaGmcqGFZoWzdaWgaaWcbaGaeGimaadabeaaaOqaaiabicdaWaqaaiabl+Uimbqaaiabl+Uimbqaaiabl+UimbqaaiabicdaWaqaaiab+r7aKnaaBaaaleaacqaIXaqmaeqaaaGcbaGaeGOmaiJae43SdC2aaSbaaSqaaiabigdaXaqabaaakeaacqaIWaamaeaacqWIVlctaeaacqWIVlctaeaacqaIWaamaeaacqaIWaamaeaacqGF0oazdaWgaaWcbaGaeGOmaidabeaaaOqaaiabikdaYiab+n7aNnaaBaaaleaacqaIYaGmaeqaaaGcbaGaeGimaadabaGaeS47IWeabaGaeSO7I0eabaGaeSO7I0eabaGaeSO7I0eabaGaeSO7I0eabaGaeSy8I8eabaGaeSO7I0eabaGaeS47IWeabaGaeS47IWeabaGaeS47IWeabaGaeGimaadabaGae4hTdq2aaSbaaSqaaiabd6gaUjabgkHiTiabigdaXaqabaaakeaacqaIYaGmcqGFZoWzdaWgaaWcbaGaemOBa4MaeyOeI0IaeGymaedabeaaaOqaaiabl+Uimbqaaiabl+Uimbqaaiabl+UimbqaaiabicdaWaqaaiabicdaWaqaaiab+r7aKnaaBaaaleaacqWGUbGBaeqaaaaaaOGaayjkaiaawMcaaaaa@7D9D@

and the entries in **M **are the probabilities of a cell in generation *i *dividing (*γ*_*i*_) or surviving without dividing (*δ*_*i*_), and *γ*_*i *_+ *δ*_*i *_≤ 1. Typically an experiment begins with a population of undivided cells and so **Z**_0 _= (*N*_0_, 0, ..., 0).

This stochastic approach also provides the covariance matrix of cell counts in each generation at time *t*, **V**_*t*_, in terms of **Z**_0_, the *E *(**Z**_*t*_) and **M **(see Methods). The framework is easily extended to calculate the quantities *E *(**Z**_*t*_) and **V**_*t *_when the parameters governing cell kinetics are also functions of time. In the analyses we present below, we used Mathematica [24] to generate *E *(**Z**_*t*_) and **V**_*t *_given initial cell counts **Z**_0 _and a set of parameters that specify a branching process model – *i.e*., how the probabilities *γ *and *δ *vary with division and/or time.

This approach can also be applied to a qualitatively different class of models, Markovian branching processes in continuous time. In these models cells have exponentially distributed lifetimes, at the end of which they either divide or die. We describe this in Appendix 1. Indeed the method we discuss in the following section applies to any stochastic model which provides the quantities *E *(**Z**_*t*_) and **V**_*t *_given a set of initial cell counts **Z**_**0**_.

#### Parameter estimation using quasi-likelihood

In principle a likelihood can be computed exactly for any branching process and a dataset. While this is feasible for small cell populations or one or two divisions, with the cell numbers encountered in most experimental situations this becomes intractable for combinatorical reasons (see Appendix 2 for a discussion). As a solution, we take a Quasi Likelihood (QL) approach which requires only the first two moments of the cell counts [25]. QL yields consistent parameter estimates, (that is, the estimates converge to their true values for large sample sizes or large numbers of cells) with minimal confidence intervals [26]. Given the large numbers of cells typically observed in experiments, one might intuitively expect that by the central limit theorem the distribution of cell counts might be well specified by their means and covariances alone.

Let the model parameters be components of the vector ***β***, at let **Y **be the observed cell counts obtained from a CFSE fluorescence profile at one time point. Let ***μ***(***β***) = *E *(**Z**_*t*_) and **V **(***β***) be respectively the expectation values and covariances of the cell counts at that timepoint, expressed as functions of the parameters. Then the following (the 'quasi score function') has properties in common with the derivative of a log-likelihood:

U(β)=DTV−1(Y−μ),where Dij∂μi∂βj.
 MathType@MTEF@5@5@+=feaafiart1ev1aaatCvAUfKttLearuWrP9MDH5MBPbIqV92AaeXatLxBI9gBaebbnrfifHhDYfgasaacH8akY=wiFfYdH8Gipec8Eeeu0xXdbba9frFj0=OqFfea0dXdd9vqai=hGuQ8kuc9pgc9s8qqaq=dirpe0xb9q8qiLsFr0=vr0=vr0dc8meaabaqaciaacaGaaeqabaqabeGadaaakeaafaqabeqacaaabaacbeGae8xvauLaeiikaGcccmGae4NSdiMaeiykaKIaeyypa0Jae8hraq0aaWbaaSqabeaacqWGubavaaGccqWFwbGvdaahaaWcbeqaaiabgkHiTiabigdaXaaakiabcIcaOiab=LfazjabgkHiTiab+X7aTjabcMcaPiabcYcaSaqaaiabbEha3jabbIgaOjabbwgaLjabbkhaYjabbwgaLjabbccaGiabbseaenaaBaaaleaacqWGPbqAcqWGQbGAaeqaaOWaaSaaaeaacqGHciITiiGacqqF8oqBdaWgaaWcbaGaemyAaKgabeaaaOqaaiabgkGi2kab9j7aInaaBaaaleaacqWGQbGAaeqaaaaaaaGccqGGUaGlaaa@5448@

These properties are *E *(**U**) = 0, cov(**U**) = **D**^*T *^**V**^-1^**D **≡ **i **(***β***) and *E *(*∂*U_*i *_(***β***)/*∂β*_*j*_) = -**i **(***β***). A QL estimator of ***β***, β * is located at a zero of **U**. The system **U **(***β***) = **0 **is a system of *r *nonlinear equations for the *r *components of the maximum QL estimate of the parameter vector β*. We use an iteratively re-weighted least squares (IRLS) algorithm, or a quasi-Newton step using Fisher scoring (that is, using the information matrix **i **as an approximation to the Hessian of **U**) to search for β* given an initial guess β0∗
 MathType@MTEF@5@5@+=feaafiart1ev1aaatCvAUfKttLearuWrP9MDH5MBPbIqV92AaeXatLxBI9gBaebbnrfifHhDYfgasaacH8akY=wiFfYdH8Gipec8Eeeu0xXdbba9frFj0=OqFfea0dXdd9vqai=hGuQ8kuc9pgc9s8qqaq=dirpe0xb9q8qiLsFr0=vr0=vr0dc8meaabaqaciaacaGaaeqabaqabeGadaaakeaaiiWacqWFYoGydaqhaaWcbaGaeGimaadabaGaey4fIOcaaaaa@305F@;

β1∗=β0∗+i0−1(β0∗)U(β0∗).
 MathType@MTEF@5@5@+=feaafiart1ev1aaatCvAUfKttLearuWrP9MDH5MBPbIqV92AaeXatLxBI9gBaebbnrfifHhDYfgasaacH8akY=wiFfYdH8Gipec8Eeeu0xXdbba9frFj0=OqFfea0dXdd9vqai=hGuQ8kuc9pgc9s8qqaq=dirpe0xb9q8qiLsFr0=vr0=vr0dc8meaabaqaciaacaGaaeqabaqabeGadaaakeaaiiWacqWFYoGydaqhaaWcbaGaeGymaedabaGaey4fIOcaaOGaeyypa0Jae8NSdi2aa0baaSqaaiabicdaWaqaaiabgEHiQaaakiabgUcaRGqabiab+LgaPnaaDaaaleaacqaIWaamaeaacqGHsislcqaIXaqmaaGccqGGOaakcqWFYoGydaqhaaWcbaGaeGimaadabaGaey4fIOcaaOGaeiykaKIae4xvauLaeiikaGIae8NSdi2aa0baaSqaaiabicdaWaqaaiabgEHiQaaakiabcMcaPiabc6caUaaa@473B@

We find convergence with this algorithm is robust to the choice of initial guess. To speed convergence, particularly with complex models, we select an initial condition by randomly generating a large sample of candidate parameter vectors and choose the one that maximises the likelihood as defined in the following section.

This estimation scheme is easily generalised to use a series of CFSE profiles obtained at multiple timepoints. This overcomes the intrinsic limitation of single CFSE timepoints, which can provide at most 8 or 9 data points, and so increases our confidence in fitted models and ability to discriminate between them. Suppose the experimental data consists of cell counts **Y**_*t *_from independent experiments at each of a set of timepoints labeled by the index *t*, and we have a model that provides the corresponding expected cell numbers ***μ***_*t *_and the covariances **V**_*t*_. Since the data at each timepoint are independent they can be used additively to construct the score function. Then if **D**_*t *_is the matrix of derivatives of the expected values ***μ***_*t *_with respect to the parameters ***β***, equation (2) holds with

i(β)=∑tDtTVt−1Dt
 MathType@MTEF@5@5@+=feaafiart1ev1aaatCvAUfKttLearuWrP9MDH5MBPbIqV92AaeXatLxBI9gBaebbnrfifHhDYfgasaacH8akY=wiFfYdH8Gipec8Eeeu0xXdbba9frFj0=OqFfea0dXdd9vqai=hGuQ8kuc9pgc9s8qqaq=dirpe0xb9q8qiLsFr0=vr0=vr0dc8meaabaqaciaacaGaaeqabaqabeGadaaakeaaieqacqWFPbqAcqGGOaakiiWacqGFYoGycqGGPaqkcqGH9aqpdaaeqbqaaiab=reaenaaDaaaleaacqWG0baDaeaacqWGubavaaaabaGaemiDaqhabeqdcqGHris5aOGae8Nvay1aa0baaSqaaiab=rha0bqaaiabgkHiTiabigdaXaaakiab=reaenaaBaaaleaacqWG0baDaeqaaaaa@4138@

and

U(β)=∑tDtTVt−1(Yt−μt).
 MathType@MTEF@5@5@+=feaafiart1ev1aaatCvAUfKttLearuWrP9MDH5MBPbIqV92AaeXatLxBI9gBaebbnrfifHhDYfgasaacH8akY=wiFfYdH8Gipec8Eeeu0xXdbba9frFj0=OqFfea0dXdd9vqai=hGuQ8kuc9pgc9s8qqaq=dirpe0xb9q8qiLsFr0=vr0=vr0dc8meaabaqaciaacaGaaeqabaqabeGadaaakeaaieqacqWFvbqvcqGGOaakiiWacqGFYoGycqGGPaqkcqGH9aqpdaaeqbqaaiab=reaenaaDaaaleaacqWG0baDaeaacqWGubavaaaabaGaemiDaqhabeqdcqGHris5aOGae8Nvay1aa0baaSqaaiab=rha0bqaaiabgkHiTiabigdaXaaakiabcIcaOiab=LfaznaaBaaaleaacqWG0baDaeqaaOGaeyOeI0Iae4hVd02aaSbaaSqaaiabdsha0bqabaGccqGGPaqkcqGGUaGlaaa@481E@

We can extend this further to deal with multiple populations present in unknown proportions, with different kinetics. Take a model in which the total initial cell numbers are known and are thought to comprise *m *distinct subpopulations, present at initial (unknown) frequencies *p*^(*i*)^. Each subpopulation labelled by index *i *then has its own expected cell numbers μt(i)
 MathType@MTEF@5@5@+=feaafiart1ev1aaatCvAUfKttLearuWrP9MDH5MBPbIqV92AaeXatLxBI9gBaebbnrfifHhDYfgasaacH8akY=wiFfYdH8Gipec8Eeeu0xXdbba9frFj0=OqFfea0dXdd9vqai=hGuQ8kuc9pgc9s8qqaq=dirpe0xb9q8qiLsFr0=vr0=vr0dc8meaabaqaciaacaGaaeqabaqabeGadaaakeaaiiWacqWF8oqBdaqhaaWcbaGaemiDaqhabaGaeiikaGIaemyAaKMaeiykaKcaaaaa@3315@ and covariances Vt(i)
 MathType@MTEF@5@5@+=feaafiart1ev1aaatCvAUfKttLearuWrP9MDH5MBPbIqV92AaeXatLxBI9gBaebbnrfifHhDYfgasaacH8akY=wiFfYdH8Gipec8Eeeu0xXdbba9frFj0=OqFfea0dXdd9vqai=hGuQ8kuc9pgc9s8qqaq=dirpe0xb9q8qiLsFr0=vr0=vr0dc8meaabaqaciaacaGaaeqabaqabeGadaaakeaaieqacqWFwbGvdaqhaaWcbaGaemiDaqhabaGaeiikaGIaemyAaKMaeiykaKcaaaaa@3292@. We construct the quantities

μt=∑ip(i)μt(i),Vt=∑ip(i)Vt(i)
 MathType@MTEF@5@5@+=feaafiart1ev1aaatCvAUfKttLearuWrP9MDH5MBPbIqV92AaeXatLxBI9gBaebbnrfifHhDYfgasaacH8akY=wiFfYdH8Gipec8Eeeu0xXdbba9frFj0=OqFfea0dXdd9vqai=hGuQ8kuc9pgc9s8qqaq=dirpe0xb9q8qiLsFr0=vr0=vr0dc8meaabaqaciaacaGaaeqabaqabeGadaaakeaafaqabeqacaaabaaccmGae8hVd02aaSbaaSqaaiabdsha0bqabaGccqGH9aqpdaaeqbqaaiabdchaWnaaCaaaleqabaGaeiikaGIaemyAaKMaeiykaKcaaOGae8hVd02aa0baaSqaaiabdsha0bqaaiabcIcaOiabdMgaPjabcMcaPaaaaeaacqWGPbqAaeqaniabggHiLdGccqGGSaalaeaaieqacqGFwbGvdaWgaaWcbaGaemiDaqhabeaakiabg2da9maaqafabaGaemiCaa3aaWbaaSqabeaacqGGOaakcqWGPbqAcqGGPaqkaaGccqGFwbGvdaqhaaWcbaGaemiDaqhabaGaeiikaGIaemyAaKMaeiykaKcaaaqaaiabdMgaPbqab0GaeyyeIuoaaaaaaa@526C@

and use these in the expressions above, with the parameter vector ***β ***now including the independent unknowns *p*^(1)^, ..., *p*^(*m *- 1)^.

The covariance matrix of the parameter estimates cov (β*) is asymptotically the inverse of the information matrix **i **(***β***). Since **U **is (asymptotically) the derivative of a log likelihood, **i**^-1 ^(***β***) is an estimate of the curvature of the log likelihood surface in parameter space. This provides confidence intervals directly if we assume no error in the cell counts **Y**_*t *_– that is, if all uncertainty in our parameter estimates comes from the underlying stochasticity of cell behaviour expressed by the model. These confidence intervals are typically rather small given the large numbers of cells usually observed in proliferation assays.

We also note that when the observations are generated by a true branching process the weighting to datapoints provided by the covariance structure is not required for generating point estimates of parameters, since the fitting procedure is essentially a minimisation of a sum of squared residuals, each of which is non-negative and is strictly zero (along with the score function) at the QL estimate of the parameters. The covariance structure is important, however, for the correct estimation of confidence intervals on branching process parameters using the information matrix, and for model discrimination using likelihood ratio tests (see below).

A Mathematica notebook which implements the calculation of the mean and covariances of the cell counts, the generation of the initial parameter estimate and the QL estimation procedure is available on request from the authors (AY and CC).

#### Model comparison

Typically there may be several candidate branching process models that might describe the biology and we want to assess the relative support for each. Again, assuming no measurement error in the observed cell counts **Y**_*t*_, the usual procedure for comparing two nested models *A *and *B*, *A *with *n *additional parameters is to use the residual deviance [25], defined as twice the difference between the maximum achievable log likelihood given the data and the log likelihood at the QL estimate of the parameters -

*D *(**Y**; ***μ***) = 2 *L *(**Y**; **Y**) - 2 *L *(**Y**; ***μ***),

where *L *(**Y**; ***μ***) is the logarithm of the likelihood of a model with expected cell counts ***μ ***generating the observations **Y**. The quantity *D*_*A *_– *D*_*B *_for models *A *and *B *is asymptotically *χ*^2^-distributed with *n *degrees of freedom. This is the standard likelihood ratio test.

The obvious approach would be to integrate the score function **U **(***β***) (eqn. (1)) to obtain an estimate of *L*. However, **U **(***β***) cannot be expressed as the gradient of a scalar function, and so the quasi-log likelihood is not uniquely specified by the parameters (see refs. [25,27] for a discussion). Instead, to compare models we propose using a log likelihood based on the generalised Pearson statistic for correlated measurements [28], which is simply the residual sum of squares weighted by the predicted covariances:

X2=∑t(Yt−μt)Vt−1(Yt−μt).
 MathType@MTEF@5@5@+=feaafiart1ev1aaatCvAUfKttLearuWrP9MDH5MBPbIqV92AaeXatLxBI9gBaebbnrfifHhDYfgasaacH8akY=wiFfYdH8Gipec8Eeeu0xXdbba9frFj0=OqFfea0dXdd9vqai=hGuQ8kuc9pgc9s8qqaq=dirpe0xb9q8qiLsFr0=vr0=vr0dc8meaabaqaciaacaGaaeqabaqabeGadaaakeaacqWGybawdaahaaWcbeqaaiabikdaYaaakiabg2da9maaqafabaGaeiikaGccbeGae8xwaK1aaSbaaSqaaiabdsha0bqabaGccqGHsisliiWacqGF8oqBdaWgaaWcbaGaemiDaqhabeaakiabcMcaPiab=zfawnaaDaaaleaacqWG0baDaeaacqGHsislcqaIXaqmaaaabaGaemiDaqhabeqdcqGHris5aOGaeiikaGIae8xwaK1aaSbaaSqaaiabdsha0bqabaGccqGHsislcqGF8oqBdaWgaaWcbaGaemiDaqhabeaakiabcMcaPiabc6caUaaa@4AF6@

The sum is over each independent timepoint and the expectation values ***μ***_*t *_and covariance matrices **V**_*t *_are evaluated at the QL parameter estimates. We note that the derivative of this quantity with respect to the parameters is the score function (1) if we neglect the terms proportional to the derivative of the covariance matrix with respect to the parameters. These terms are second order in the difference between the data and the QL prediction provided by the model. We then calculate a 'surrogate' log likelihood ℒ
 MathType@MTEF@5@5@+=feaafiart1ev1aaatCvAUfKttLearuWrP9MDH5MBPbIqV92AaeXatLxBI9gBaebbnrfifHhDYfgasaacH8akY=wiFfYdH8Gipec8Eeeu0xXdbba9frFj0=OqFfea0dXdd9vqai=hGuQ8kuc9pgc9s8qqaq=dirpe0xb9q8qiLsFr0=vr0=vr0dc8meaabaqaciaacaGaaeqabaqabeGadaaakeaat0uy0HwzTfgDPnwy1egaryqtHrhAL1wy0L2yHvdaiqaacqWFsectaaa@376D@ using the relation

ℒ=−12X2
 MathType@MTEF@5@5@+=feaafiart1ev1aaatCvAUfKttLearuWrP9MDH5MBPbIqV92AaeXatLxBI9gBaebbnrfifHhDYfgasaacH8akY=wiFfYdH8Gipec8Eeeu0xXdbba9frFj0=OqFfea0dXdd9vqai=hGuQ8kuc9pgc9s8qqaq=dirpe0xb9q8qiLsFr0=vr0=vr0dc8meaabaqaciaacaGaaeqabaqabeGadaaakeaat0uy0HwzTfgDPnwy1egaryqtHrhAL1wy0L2yHvdaiqaacqWFsectcqGH9aqpcqGHsisldaWcaaqaaiabigdaXaqaaiabikdaYaaacqWGybawdaahaaWcbeqaaiabikdaYaaaaaa@3DAA@

=−12∑t(Yt−μt)Vt−1(Yt−μt).
 MathType@MTEF@5@5@+=feaafiart1ev1aaatCvAUfKttLearuWrP9MDH5MBPbIqV92AaeXatLxBI9gBaebbnrfifHhDYfgasaacH8akY=wiFfYdH8Gipec8Eeeu0xXdbba9frFj0=OqFfea0dXdd9vqai=hGuQ8kuc9pgc9s8qqaq=dirpe0xb9q8qiLsFr0=vr0=vr0dc8meaabaqaciaacaGaaeqabaqabeGadaaakeaacqGH9aqpcqGHsisldaWcaaqaaiabigdaXaqaaiabikdaYaaadaaeqbqaaiabcIcaOGqabiab=LfaznaaBaaaleaacqWG0baDaeqaaOGaeyOeI0cccmGae4hVd02aaSbaaSqaaiabdsha0bqabaGccqGGPaqkcqWFwbGvdaqhaaWcbaGaemiDaqhabaGaeyOeI0IaeGymaedaaaqaaiabdsha0bqab0GaeyyeIuoakiabcIcaOiab=LfaznaaBaaaleaacqWG0baDaeqaaOGaeyOeI0Iae4hVd02aaSbaaSqaaiabdsha0bqabaGccqGGPaqkcqGGUaGlaaa@4B73@

This is essentially a multivariate normal approximation to the true log likelihood.

To compare non-nested models, the simplest approach is to compare the absolute values of likelihoods (see, for example, [20]) or to use the Akaike Information Criterion. This is necessary when comparing the fits with different timesteps, of which there are usually a restricted set of discrete choices; these are dictated by the maximum division number observed at each timepoint, and the intervals between these timepoints. It can also be used to compare members of a family of models with the same number of parameters – for example, when division or death probabilities are assumed to change at a given, but unknown, division number.

### 2. Validation of the method

#### Testing the validity of the QL estimator

**Figure 2:**
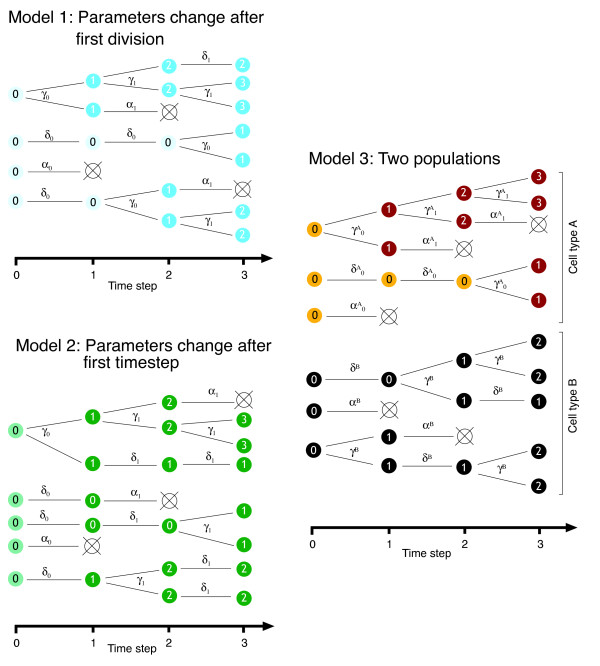
**Validation of the quasi-likelihood estimation procedure with artificial datasets**. We generated simulated CFSE datasets using numerical realisations of three different branching processes models of cell kinetics, and tested our estimation procedure by using these datasets to estimate the model parameters. As in Figure 1, division probabilities are represented by γ, survival without division as *δ*, and death as *α* = 1 - *γ *- *δ.***Model 1** – division and death probabilities change after the first division. Changes in parameters are indicated by different shading of cells. **Model 2** – Probabilities of division and death change after one timestep. **Model 3** – Resolving two subpopulations. We generated artificial CFSE profiles by adding the contributions from two branching processes – one with cell type A, in which division and death probabilities changed after first division, and one with cell type B, with constant probabilities of division and death. Type A cells were present at initial frequency f_A_. For each Model (1, 2, 3) we generated time series of simulated CFSE data sets by running three independent branching processes (each starting with 10_4_ cells) and used the counts in each generation after 2, 4 and 6 timesteps as independent timepoints. This ensured that the data at each timestep were uncorrelated measurements and so would contribute additively to the log likelihood. 10_4_ replicate timeseries were generated for each model and used with the QL procedure to estimate the parameters.

A condition for consistency and normality of the QL estimate β* is that cell numbers in all generations are large. As a preliminary test of the method, and to confirm that QL estimates are reliable when used with the numbers of cells encountered in experimental situations, we used a Monte Carlo procedure to examine the properties of the estimator. We generated synthetic CFSE profiles with repeated numerical simulations of branching processes with three different models, each starting with 10^4 ^cells. These cell numbers are lower than those typically used in proliferation assays. The models are described in detail in Figure 2 (also see table 1). In model 1, parameters change after the first division; in model 2, the parameters change after the first timestep, and in model 3 we include two populations, one with division-dependent probabilities of division and death, and the other with constant probabilities. For each simulation we calculated the QL estimate of the parameters and their associated confidence intervals assuming asymptotic normality. We then calculated the proportion of simulations in which the predicted confidence intervals contained the true value of each parameter. The close agreement of true and estimated parameters and the accuracy of the predicted 95% and 99% confidence intervals validates our use of QL to estimate parameters with large populations of cells.

**Figure 3:**
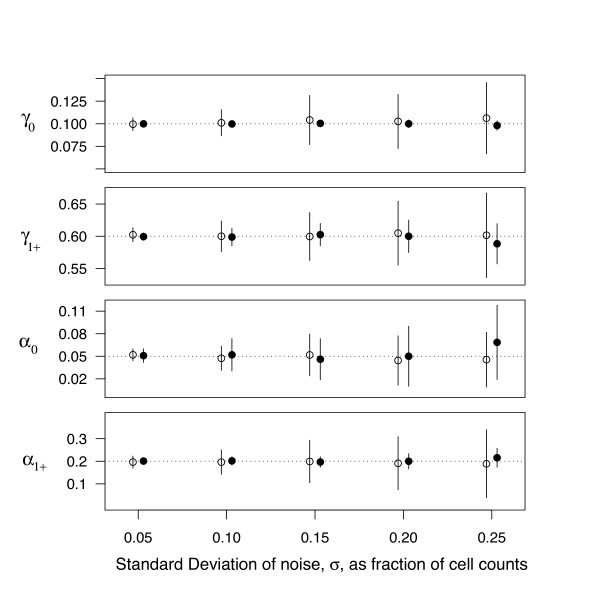
**Quasi-likelihood estimation in the presence of noise**. Synthetic CFSE datasets were generated with branching process Model 1, in which probabilities of division and death change after the first division; parameter values were *γ*_0 _= 0.1, *γ*_1+ _= 0.6, *α*_0 _= 0.05, *α*_1+ _= 0.2, starting with 10^6 ^cells. QL estimation was used to identify all four best-fit parameter values from a single timepoint – the counts in generations 0–6 observed after 6 timesteps – as increasing levels of Gaussian noise were added either to the counts in each generation (open circles) or the total cell counts, keeping the proportions of cells in each generation constant (filled circles). Noise level *σ *indicates that the cell counts (or total numbers) were multiplied by a factor (1 + *ε*) where *ε *is a random number drawn from *N *(0, *σ*^2^)). We show the mean and standard deviation of 100 simulations. Dotted horizontal lines indicated the true values of the parameters.

#### Validation of the method in the presence of measurement noise

As a more stringent test we examined how well the QL method could recover branching process parameters in the presence of measurement error (Figure 3). Using model 1 (in which division and death probabilities per timestep changed after the first division) we again used simulated branching processes to generate multiple realisations of a single CFSE timepoint, comprising the cell numbers in 6 generations after 5 timesteps. We then added Gaussian noise of varying amplitudes to (i) the cell counts in each generation (Figure 3, open circles), or (ii) the total cell count (filled circles), preserving the proportions of the population in each generation. The latter scenario is commonly encountered in *in vivo *studies in which recovered cell numbers may be subject to significant uncertainty but the frequencies of cells in each CFSE peak may show little variation between experiments.

**Figure 4:**
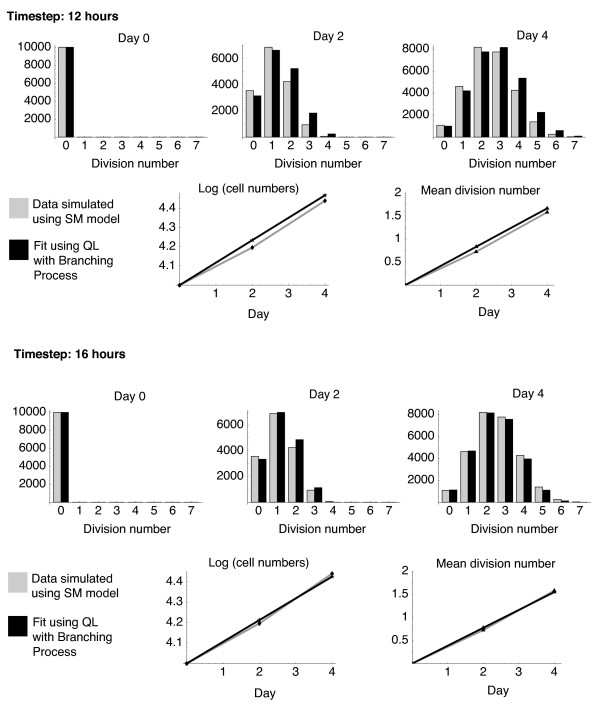
**Using branching processes to describe data generated with the Smith-Martin model of cell kinetics**. Fitting discrete-time branching process (BP) models to a dataset generated with the homogeneous Smith-Martin (SM) model. The dataset comprise 10_4_ cells in the A phase at time zero, and the total numbers in each generation (i.e. in both A and B phases) at days 2 and 4. We used SM parameter values *λ* = 0.5, *Δ* = 1/3 day (8 hours) and *μ* = 0.1. Two choices of uniform timestep gave reasonable fits – 12 hours (upper panels) and 16 hours (lower panels). We fitted several branching process models for all choices of timestep and in each case the best fit was a homogeneous model with constant probabilities of division and death. The 16 hour timestep gave the best fit (log likelihood (12h timestep) =426; -log likelihood (16h timestep) = 112), with *γ* = 0.239 and *α* = 0.064 being the estimated probabilities of division and death in each 16 h time interval respectively.

We make three simple observations here. First, the uncertainty in parameters scales approximately linearly with the amplitude of the noise, and a given fractional uncertainty *σ *in cell counts translates into a comparable fractional uncertainty in parameter estimates. Second, the division probabilities strongly in fluence the shape of the CFSE profile and so in general are estimated more accurately when total counts are subject to noise than when cell counts in each generation are subject to independent error. Third, the division and death probabilities that apply to more CFSE peaks or measurements (in this example, *γ*_1+ _and *α*_1+_, which determine the division and death probabilities for all cells in generations 1 and above) can be estimated more accurately than those constrained by fewer measurements (here, *γ*_0 _and *α*_0 _for undivided cells). This effect is again more pronounced when the proportions of cells in each generation are known more accurately than the total numbers.

#### Relation of parameters to more complex models

As described in the introduction, the branching process is perhaps a minimal description of cell kinetics. To investigate how and under what conditions its parameters can be related to those of more detailed models, we used synthetic CFSE datasets generated with the homogeneous Smith-Martin model. In this model cells spend exponentially distributed times in the A-phase (G0/G1), with mean 1/*λ*. Cells triggered to divide then transit through a B-phase (S/G2/M) with duration Δ before generating two daughter cells and returning to the A-phase. We assume death is independent of division and occurs at rate *μ *in both A-or B-phases. In Figure 4 we show that the QL procedure identifies a homogeneous model as the best description of the data.

**Figure 5:**
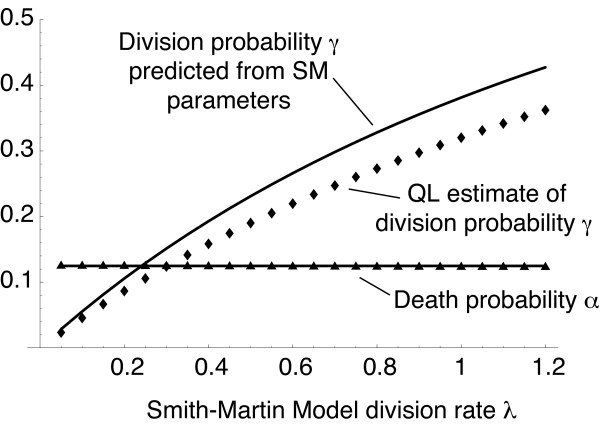
**Relating parameters in branching process and Smith-Martin models**. Synthetic CFSE datasets were generated using the homogeneous Smith-Martin model with different division rates *λ *and *μ *= 0.2 day^-1 ^and Δ = 12 hours. Each dataset contained the cell numbers in each generation at days 2 and 4. Branching process (BP) models were fitted to each. In all cases the best fit was provided by a homogeneous branching process with a timestep of 16 hours, as measured by the absolute value of the log likelihood. The QL estimates of the division probabilities *γ *are shown as diamonds, and the death probabilities *α *as triangles. Predicted values using the approximations – eqns (5) and (6) – are shown as solid lines.

The parameters in the branching process (BP) and Smith-Martin (SM) models can be related with some approximations. In this instance of the SM model the probability of a cell dying during a finite interval *τ*, the branching process parameter *α*, is independent of the cell being in the A or B phase and so we predict that the QL estimate *α *should be given by

*α *= 1 – *e*^-*μτ*^.

To divide during an interval *τ*, a cell must complete a B-phase during that interval. If Δ <*τ *< 2 Δ, the expected proportion of cells to divide and survive is approximately

γ≃λ−1λ−1+Δ︸Fraction in A(1−e−λ(τ−Δ))e−μτ+Δλ−1+Δ︸Fraction in Be−μτ
 MathType@MTEF@5@5@+=feaafiart1ev1aaatCvAUfKttLearuWrP9MDH5MBPbIqV92AaeXatLxBI9gBaebbnrfifHhDYfgasaacH8akY=wiFfYdH8Gipec8Eeeu0xXdbba9frFj0=OqFfea0dXdd9vqai=hGuQ8kuc9pgc9s8qqaq=dirpe0xb9q8qiLsFr0=vr0=vr0dc8meaabaqaciaacaGaaeqabaqabeGadaaakeaafaqabeGabaaabaacciGae83SdCMaeS4qISZaaGbaaeaadaWcaaqaaiab=T7aSnaaCaaaleqabaGaeyOeI0IaeGymaedaaaGcbaGae83UdW2aaWbaaSqabeaacqGHsislcqaIXaqmaaGccqGHRaWkcqqHuoaraaaaleaacqqGgbGrcqqGYbGCcqqGHbqycqqGJbWycqqG0baDcqqGPbqAcqqGVbWBcqqGUbGBcqqGGaaicqqGPbqAcqqGUbGBcqqGGaaicqqGbbqqaOGaayjo+dWaaeWaaeaacqaIXaqmcqGHsislcqWGLbqzdaahaaWcbeqaaiabgkHiTiab=T7aSjabcIcaOiab=r8a0jabgkHiTiabfs5aejabcMcaPaaaaOGaayjkaiaawMcaaiabdwgaLnaaCaaaleqabaGaeyOeI0Iae8hVd0Mae8hXdqhaaaGcbaGaaCzcaiabgUcaRmaayaaabaWaaSaaaeaacqqHuoaraeaacqWF7oaBdaahaaWcbeqaaiabgkHiTiabigdaXaaakiabgUcaRiabfs5aebaaaSqaaiabbAeagjabbkhaYjabbggaHjabbogaJjabbsha0jabbMgaPjabb+gaVjabb6gaUjabbccaGiabbMgaPjabb6gaUjabbccaGiabbkeacbGccaGL44pacqWGLbqzdaahaaWcbeqaaiabgkHiTiab=X7aTjab=r8a0baaaaaaaa@7F82@

We tested the validity of the approximations (5) and (6) by fitting BP models to a series of datasets generated by varying the division rate *λ *in the SM model. For each we compared the quasi-likelihood estimates of the BP parameters *γ *and *α *with their approximations. The results are shown in Figure 5.

**Figure 6:**
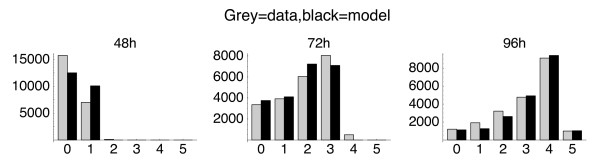
**Estimating parameters from T cell proliferation data**. The best fit of a heterogeneous discrete-time branching process model to a CFSE timecourse obtained by *in vitro *stimulation of 2.5 × 10^4 ^human CD8^+ ^T cells with anti-CD3 and anti-CD28 in saturating quantities of IL-2. *γ *refers to division probability, *α *to death. Cells from independent cultures were recovered and analysed for CFSE content at 24 h intervals after stimulation. The histograms show total cell numbers in each generation (grey bars, observed counts obtained by the EM algorithm; black bars, predicted counts with best-fit branching process model). The best fit model gave a timestep of 12 h and indicated that division (*γ*) and death (*α*) probabilities changed with generation number (Table 2).

The QL procedure identifies the homogeneous model correctly and the estimated death probability *α *agrees closely with the predicted value for all division rates. The QL estimate of division probability *γ *agrees well with the predicted value (6) when the SM division rate *λ *is low, but the two diverge as *λ *increases. The discrete time process does not specify the true (continuous) distribution of interdivision times, but instead 'coarse-grains' this distribution by allowing division at any time within each timestep. For constant probabilities of division and death, this generates a geometric distribution in discrete time, such that (in the absence of cell death) the probability that a given cell observed since *t *= 0 divides during the interval *t*' = *nτ *and *t *= (*n *+ 1) *τ *is *P *(*n*) = *γ *(1 - *γ*)^*n*^; while for the SM model with constant parameters the probability density for the interdivision time *t*, *P *(*t*), is exponential with a delay, or *P *(*t*) = 0 for 0 ≤ *t *≤ Δ and *P *(*t*) = *λ *exp (-*λ *(*t *- Δ)) for *t *> Δ. These distributions converge for *t *= *nτ *when division rates are low; that is, when the timestep *τ *is smaller than the average time spent in the A-phase (*τ *<< 1/*λ*) and when the average time spent in the A-phase is much longer than the B-phase (11/*λ *>> Δ).

### 3. Dealing with experimental CFSE data

An important issue when quantifying the dynamics of CFSE-labeled cells is assessing our confidence in the observed cell counts **Y**_*t*_. In this section we discuss how to deal with various sources of uncertainty in the cell counts and how these impact on model fitting and comparison. Another significant source of disagreement between model and observations, of course, is that the underlying model may not represent the biology well. With this in mind, what we discuss here applies not only to the discrete time branching models we describe here but also to any stochastic model of cell division that can be used to provide likelihood-based parameter estimates.

#### Uncertainties in the assignment of cells to generations from CFSE profiles

The process of assigning a division number to cells in a CFSE profile can be a significant source of error, particularly if the peaks corresponding to cells in one generation are ill-defined. The distributions of neighbouring peaks usually overlap significantly, and cells in the tails of these distributions may be mis-assigned to neighbouring generations. Further, the factor difference in median fluorescence intensity of adjacent peaks is typically not exactly 2, and this error can amount to uncertainties of as much as a whole division for cells that have divided multiple times. This is particularly noticeable in CFSE profiles which contain distinct subpopulations of cells separated by several divisions and with few cells to mark the location of intermediate generations. In many circumstances, then, the 'gating' or assignment of cells to different divisions is itself a process of inference.

We used a standard algorithm to perform this, based on the Expectation-Maximisation (EM) algorithm [29]. EM is a bounded optimisation technique for the computation of maximum likelihoods typically used in incomplete-data problems. CFSE histograms generated in experiments (*i.e*., the plot of event counts against the logarithm of fluorescence intensity) can usually be approximated well by normal mixtures (*i.e*. a superposition of Gaussian distributions) and estimating the parameters for such a normal mixture is a standard application of the EM algorithm. In practice, we find that the algorithm works well only if we provide good initial conditions for the modes (maxima) of each normal component in the mixture, as well as some constraint on the variance of each component. Initial locations for modes are found by first specifying the data range which contains 99% of the total events, then calculating the offset (alignment of entire fit) and stride (the average fold reduction in fluorescent intensity between peaks) that produce the average largest event count. This works well because the inter-peak distances for CFSE profiles tend to be similar, as we would expect if CFSE is equally distributed between daughter cells. As a result, the initial modes are regularly spaced; however, the EM algorithm is then free to adjust the modes to produce the best fit. We heuristically set a constraint such that the variance of each component is less than or equal to that of the component with the tallest peak. Counts are then estimated using the relative area under each normal component scaled by the total number of cells.

We propose that the uncertainty in the assignment of cells to divisions can be used with a Monte Carlo procedure to assign confidence intervals to maximum-likelihood model parameter estimates from a single CFSE dataset. The method is as follows.

1. Use the EM method to identify a maximum-likelihood set of log-normal profiles from a raw CFSE profile containing *N*_0 _cells. We refer to the resulting set of counts of cells in each generation as **Y**^(0)^, where the sum of the elements of **Y**^(0) ^equals *N*_0_.

2. Using **Y**^(0) ^and a model characterised by a set of parameters ***β***, calculate a best-fit (QL) set of parameter estimates ***β***_0_.

3. Generate *P *artificial CFSE profiles, as follows. For each generation or peak *k *in the original profile, draw Yk(0)
 MathType@MTEF@5@5@+=feaafiart1ev1aaatCvAUfKttLearuWrP9MDH5MBPbIqV92AaeXatLxBI9gBaebbnrfifHhDYfgasaacH8akY=wiFfYdH8Gipec8Eeeu0xXdbba9frFj0=OqFfea0dXdd9vqai=hGuQ8kuc9pgc9s8qqaq=dirpe0xb9q8qiLsFr0=vr0=vr0dc8meaabaqaciaacaGaaeqabaqabeGadaaakeaaieaacqWFzbqwdaqhaaWcbaGaem4AaSgabaGaeiikaGIaeGimaaJaeiykaKcaaaaa@3218@ random numbers from the log-normal probability distribution used to fit that peak. This generates a population of *N*_0 _cells with fluorescent intensities drawn from the predicted distributions. Use this to re-estimate the numbers of cells in each division using the EM method. Repeat this *P *times. This generates a set of new, artificial CFSE fluorescence profiles (**Y**^(1)^, **Y**^(2)^, ..., **Y**^(*P*)^) derived from the original counts **Y**^(0)^.

4. For each artificial dataset **Y**^(*i*) ^calculate a parameter set estimate ***β***_*i*_.

5. We now have *P *samples from a probability distribution of parameter estimates representing our uncertainty in the assignment of division numbers to cells in the original CFSE profile. Calculate confidence limits on ***β***_0 _from this distribution.

As noted above, if the procedure provides estimates of the division and quiescence probabilities *γ *and *δ*, probabilities of death *α *can be calculated using *α *= 1 - *γ *- *δ*. It is then straightforward to calculate confidence intervals on *α *given the distribution of estimates of *γ *and *δ*.

We also note that each estimate ***β***_*i *_comes with its own confidence limits, stemming from the stochasticity of the branching process. We thus have at least two independent sources of uncertainty in parameters – one that stems from the uncertainty in the assignment of cells to different generations, which we estimate with the Monte Carlo procedure above; and the other from the underlying stochasticity of the branching processes – that is the range of parameter values that could reasonably (*i.e*. with some significant probability) have generated each of the datasets (**Y**^(0)^, **Y**^(1)^, ..., **Y**^(*P*)^).

This procedure assumes high levels of confidence in the measured total cell numbers. If only a single experimental replicate is available, one may have little *a priori *knowledge of the uncertainty in total cell counts and its effect on parameter estimates. This may be significant in *in vitro *experiments, but is particularly important when tracking CFSE-labeled cells *in vivo*. For example, if labeled cells are transferred to an animal and recovered blood and/or lymphoid tissues at a later timepoint, there may be both loss of cells in the recovery procedure as well as uncertainties in the number transferred successfully (*e.g*. the initial 'take' after intravenous transfer). We suggest that in the absence of experimental replicates, one approach to this problem is to make a heuristic estimate of the error in total counts, and then apply noise at this level to the total cell counts in the Monte Carlo procedure described above. We describe this in the example that follows.

#### Application to an experimental dataset

To illustrate our method of estimation with branching processes, we apply it to an experimental CFSE dataset (Figure 6). We modeled the response of a polyclonal population of CD8^+ ^T cells to stimulation *in vitro *with anti-CD3 and and anti-CD28 antibodies, in the presence of IL-2 (a growth factor). CFSE profiles from independent cultures were obtained at days 1–4. Little cell death or division was observed in the first 24 h so the 24 h timepoint was taken as the initial condition. The majority of T cells were expected to respond to this stimulus and so we modeled the system as a single population with division or death parameters varying (possibly) with time and/or generation number.

We fitted a variety of models to this data, allowing parameters to vary with time and/or division. The optimal timestep for all models (as measured by the absolute value of the likelihood) was 12 h, and assuming no divisions took place before 36 h. A reasonable fit was obtained with a four-parameter model that allowed undivided cells (generation 0) and divided cells (generations 1+) to have distinct probabilities of division and death; an extension to six parameters allowed different division and death probabilities in generations 0, 1–3 and 4+. The extended model gave a significantly better fit (*χ*^2 ^test on the difference in log likelihoods, on 2 degrees of freedom, *p *< 10^-6^). The best fit using the six-parameter model and the corresponding parameter estimates are shown in Figure 6 and Table 2.

These results suggest slow recruitment of undivided cells into division after 36 hours, with a significant probability of apoptosis in the undivided population. Cells that have divided once divide again with approximately 40% probability in each 12 h interval, with increased susceptibility to apoptosis; division slows significantly in the fourth generation. Thus the method identifies the slow first division commonly observed in T cell proliferation assays; it also suggests that cells dividing rapidly have an associated high probability of death.

We quote confidence intervals on the parameter estimates using (i) the asymptotic properties of the QL estimator; (ii) the Monte Carlo (MC) method, taking into account the uncertainty of assigning cells to CFSE peaks, and (iii) the more conservative MC method, applying an additional estimate of measurement error (5% Gaussian noise applied to total cell numbers) to each of the MC replicates. We note that the parameters governing the 4th division are not well constrained as their estimation depends on the single measurement of the cell counts in generation 5 at 96 h.

#### Comparing models using estimation of measurement error

An alternative approach with single experimental datasets is to incorporate a contribution **Λ **to the covariance matrices **V**_*t *_which represents the combined effects of our uncertainty in the assignment of generation numbers to cells and in total cell counts. The noise is then described by parameters to be estimated directly, and can be considered in the comparison of the fit of different models. Perhaps the simplest reasonable form for **Λ **is

Λ=(σρ0⋯⋯ρσρ⋯⋯0ρ⋱⋯⋯⋯⋯⋯σρ⋯⋯⋯ρσ),
 MathType@MTEF@5@5@+=feaafiart1ev1aaatCvAUfKttLearuWrP9MDH5MBPbIqV92AaeXatLxBI9gBaebbnrfifHhDYfgasaacH8akY=wiFfYdH8Gipec8Eeeu0xXdbba9frFj0=OqFfea0dXdd9vqai=hGuQ8kuc9pgc9s8qqaq=dirpe0xb9q8qiLsFr0=vr0=vr0dc8meaabaqaciaacaGaaeqabaqabeGadaaakeaaiiqacqWFBoatcqGH9aqpdaqadaqaauaabeqafuaaaaaabaacciGae43WdmhabaGae4xWdihabaGaeGimaadabaGaeS47IWeabaGaeS47IWeabaGae4xWdihabaGae43WdmhabaGae4xWdihabaGaeS47IWeabaGaeS47IWeabaGaeGimaadabaGae4xWdihabaGaeSy8I8eabaGaeS47IWeabaGaeS47IWeabaGaeS47IWeabaGaeS47IWeabaGaeS47IWeabaGae43WdmhabaGae4xWdihabaGaeS47IWeabaGaeS47IWeabaGaeS47IWeabaGae4xWdihabaGae43WdmhaaaGaayjkaiaawMcaaiabcYcaSaaa@5E13@

where the next-to-diagonal elements *ρ *represent the misassignment between generations, and the diagonal elements *σ *represent the combination of misassignment and error in total cell counts, if any. The matrix **Λ **may also be expected to vary between timepoints (**Λ **= **Λ**_*t*_). We refer to the parameters that characterize these matrices collectively as ***η***.

We cannot apply our QL procedure as it stands to estimate these additional parameters, since they do not appear in the expressions for the expected values of cell counts. Instead, we suggest that the entire parameter set (***β*, *η***) might be estimated by direct maximisation of a full multivariate normal approximation to the log likelihood,

ℒ(β,η)=−12∑t,ilog⁡det⁡ (Vt(β)+Λt(η))+(Yt(i)−μt(β))(Vt(β)+Λt(η))−1(Yt(i)−μt(β)),
 MathType@MTEF@5@5@+=feaafiart1ev1aaatCvAUfKttLearuWrP9MDH5MBPbIqV92AaeXatLxBI9gBaebbnrfifHhDYfgasaacH8akY=wiFfYdH8Gipec8Eeeu0xXdbba9frFj0=OqFfea0dXdd9vqai=hGuQ8kuc9pgc9s8qqaq=dirpe0xb9q8qiLsFr0=vr0=vr0dc8meaabaqaciaacaGaaeqabaqabeGadaaakeaafaqabeGabaaabaWenfgDOvwBHrxAJfwnHbqeg0uy0HwzTfgDPnwy1aaceaGae8NeHWKaeiikaGcccmGae4NSdiMaeiilaWIae43TdGMaeiykaKIaeyypa0JaeyOeI0YaaSaaaeaacqaIXaqmaeaacqaIYaGmaaWaaabeaeaacyGGSbaBcqGGVbWBcqGGNbWzcyGGKbazcqGGLbqzcqGG0baDaSqaaiabdsha0jabcYcaSiabdMgaPbqab0GaeyyeIuoakiabbccaGiabcIcaOGqabiab9zfawnaaBaaaleaacqWG0baDaeqaaOGaeiikaGIae4NSdiMaeiykaKIaey4kaSccceGaeW3MdW0aaSbaaSqaaiabdsha0bqabaGccqGGOaakcqGF3oaAcqGGPaqkcqGGPaqkcqGHRaWkaeaadaqadaqaaiab9LfaznaaDaaaleaacqWG0baDaeaacqGGOaakcqWGPbqAcqGGPaqkaaGccqGHsislcqGF8oqBdaWgaaWcbaGaemiDaqhabeaakiabcIcaOiab+j7aIjabcMcaPaGaayjkaiaawMcaaiabcIcaOiab9zfawnaaBaaaleaacqWG0baDaeqaaOGaeiikaGIae4NSdiMaeiykaKIaey4kaSIaeW3MdW0aaSbaaSqaaiabdsha0bqabaGccqGGOaakcqGF3oaAcqGGPaqkcqGGPaqkdaahaaWcbeqaaiabgkHiTiabigdaXaaakmaabmaabaGae0xwaK1aa0baaSqaaiabdsha0bqaaiabcIcaOiabdMgaPjabcMcaPaaakiabgkHiTiab+X7aTnaaBaaaleaacqWG0baDaeqaaOGaeiikaGIae4NSdiMaeiykaKcacaGLOaGaayzkaaGaeiilaWcaaaaa@9008@

where now the sum is over all timepoints *t *and over all replicates (Monte Carlo or experimental), *i*.

This quantity can be used directly for model comparison, either with likelihood ratio tests or information criteria statistics such as the AIC [30], although obtaining the estimate of ℒ
 MathType@MTEF@5@5@+=feaafiart1ev1aaatCvAUfKttLearuWrP9MDH5MBPbIqV92AaeXatLxBI9gBaebbnrfifHhDYfgasaacH8akY=wiFfYdH8Gipec8Eeeu0xXdbba9frFj0=OqFfea0dXdd9vqai=hGuQ8kuc9pgc9s8qqaq=dirpe0xb9q8qiLsFr0=vr0=vr0dc8meaabaqaciaacaGaaeqabaqabeGadaaakeaat0uy0HwzTfgDPnwy1egaryqtHrhAL1wy0L2yHvdaiqaacqWFsectaaa@376D@ by numerical maximisation of (7) may be difficult for complex models. This has the flavour of a mixed-effects approach [31,32] in which the original 'fixed' effects represented by the quantities ***μ***_*t *_(***β***) and **V**_*t *_(***β***) are augmented by the 'random' effects **Λ**. The parallel remains to be explored further, however, as in our case random effects are represented at the level of the variance in the predicted response ***μ ***rather than in the underlying parameters ***β ***as in standard mixed-effects models. The estimation of additional parameters in the variance function has previously been discussed as an extension to the quasi-likelihood method [33], but the non-integrability of the score function that we note above prevents the use of this formalism directly.

#### Estimation of timestep

A natural choice of timestep for single CFSE measurements is provided by the number of divisions observed during the experiment. That is, if it is clear that cells have undergone at most *n *divisions times over a time *t*, this suggests a timestep of *t/n*.

The timesteps are not required to be of equal length, although dividing the duration of the experiment into equal intervals provides the most intuitive interpretation of the probabilities of division and death per time-step as 'rates' for these processes. The method we use here is to generate a discrete set of candidate timesteps that are consistent with the number of significant peaks observed at each timepoint in a CFSE dataset, and simply search systematically for the combination of model and timestep that maximises the (quasi-) likelihood.

For some choices of timestep, however, the model may predict peaks that are not observed experimentally. For example, cells that have divided more than 8–9 times become CFSE-negative and rates of division may be underestimated by neglecting them. Peaks at the extremes of the CFSE profile may also be difficult to resolve. A particular choice of timestep might predict an additional small population of cells beyond the highest observed division number, or that some cells may have divided beyond the limits of CFSE detectability; if this timestep otherwise appears to provide a good description of the data, one might wish to include it in the set of candidates. In this case, we propose another use of the EM method to reconstruct this 'missing' data and generate an appropriate estimate of the likelihood. To take an example, suppose a model predicts that *n *+ 1 divisions should be observed at time *t *but that we can only confidently identify cells in generations 0-*n*. Choose a timestep of *t*/(*n *+ 1) and use the following iterative procedure to estimate parameters:

1. Start with a dataset that contains zero cells in generation *n *+ 1.

2. Generate QL parameter estimates using this dataset.

3. Calculate the expected numbers of cells in the 'missing' peak using these parameter values and construct a new dataset with this number of cells in generation *n *+ 1.

4. Repeat steps 2 and 3 until parameter estimates converge.

## Discussion

In this paper we have presented a method for fitting and comparing classical branching process models of cell division and death to data from CFSE labeling experiments. All parameters of this class of models can be estimated from CFSE data alone. To do this, we take a Quasi-Likelihood approach, overcoming the problem of non-computability of the exact likelihood. Further, by modeling explicitly the different sources of uncertainty in present in CFSE data, the methods we describe here can improve on existing approaches to estimating parameters, which use least squares fitting with the assumption that cell counts in each generation are subject to errors of constant variance.

Many other approaches to modeling CFSE data characterise the continuous distribution of interdivision times explicitly (exponential for simple ODE models, delayed exponential for Smith-Martin models, lognormal for the first division in the model used by Gett and Hodgkin). In contrast, the discrete-time branching process models we discuss here deal only with the average probabilities of division or death during a finite time interval. While this might be seen as a limitation, in many cases the true distribution of interdivision times may be unknown and the discrete-time approach may provide more robust predictions than with other models. The discrete timestep also provides a lower bound on the time taken for a cell to divide without modeling transit through the cell cycle explicitly, and the parameters of these models are identifiable given sufficient CFSE data. We suggest that the branching process approach is particularly suitable for analysis of data in which prior information regarding division kinetics is limited, as well as providing a method of dealing simultaneously with stochastic fluctuations and measurement errors.

Differences in the expected cell counts predicted by any model and the data, assuming the model is a faithful representation of the cell dynamics, stem from (1) the contributions of stochastic fluctuations (if any) from the model and (2) other forms of experimental noise. In the limit where the contribution of (2) overwhelms that from (1), we suggest the Monte Carlo method described here can be used to estimate confidence intervals on model parameters, and that any covariance structure predicted by the stochastic model can reasonably be neglected and only the expectation values need be used. In this case, proper model comparison using the likelihood, which explicitly contains the weightings (variances) associated with each CFSE peak, becomes very dependent on reasonable estimates being obtained for these weights. These are best estimated simply and empirically with replicate datasets. On the other hand, if cell numbers are small and and measurement errors are smaller than or comparable to fluctuations, or when only a single replicate is available, the covariance structure is important as a basis for inference.

By using knowledge of initial cell numbers and total cell counts at subsequent timepoints, models applied to CFSE data allow the estimation of death rates (averaged over all phases of the cell cycle) without the requirement of an assay for dead cells. This is particularly useful as dead cells do not persist in culture for long, and are particularly difficult to identify *in vivo*, so direct estimates of their numbers are error-prone. However, a limitation of all current methods of estimating death rates from CFSE alone is that cells may remain CFSE-positive for a short time after death and be counted as live. To improve the reliability of these estimates, for example, cells can be co-stained with Propidium Iodide to exclude those whose DNA content has been degraded.

We note that the moment-based QL estimation procedure can be applied to any stochastic model of cell dynamics which provides a covariance structure, and is not restricted to branching processes. We also emphasise that the Monte Carlo procedure for quantifying errors in the counts derived from CFSE fluorescence profiles can be applied directly to parameter estimation with deterministic models. Whatever description of the dynamics is used, treating the different sources of uncertainty in cell population data in the way we describe here allows us to more carefully test and discriminate between models of cell dynamics.

## Methods

### Detailed derivation of the moments of the distribution of cell counts

Here we show the calculation of moments of the probability distribution of the cell counts **Z**_*t *_for a stationary branching process, one in which the probabilities *δ*_*i *_and *γ*_*i *_are independent of time. We use a probability-generating function (pgf) approach.

To illustrate the use of a pgf, first consider a very simple (single-type) branching process in discrete time, which models the total number of cells in a population that is dividing and dying stochastically, and does not distinguish cells by generation. In each timestep every cell can do one of three things: divide, die or survive without dividing. These possibilities can be represented with the following pgf,

f(s)≡∑ipisi≡(1−γ−δ)+δs+γs2,
 MathType@MTEF@5@5@+=feaafiart1ev1aaatCvAUfKttLearuWrP9MDH5MBPbIqV92AaeXatLxBI9gBaebbnrfifHhDYfgasaacH8akY=wiFfYdH8Gipec8Eeeu0xXdbba9frFj0=OqFfea0dXdd9vqai=hGuQ8kuc9pgc9s8qqaq=dirpe0xb9q8qiLsFr0=vr0=vr0dc8meaabaqaciaacaGaaeqabaqabeGadaaakeaacqWGMbGzcqGGOaakcqWGZbWCcqGGPaqkcqGHHjIUdaaeqbqaaiabdchaWnaaBaaaleaacqWGPbqAaeqaaOGaem4Cam3aaWbaaSqabeaacqWGPbqAaaaabaGaemyAaKgabeqdcqGHris5aOGaeyyyIORaeiikaGIaeGymaeJaeyOeI0ccciGae83SdCMaeyOeI0Iae8hTdqMaeiykaKIaey4kaSIae8hTdqMaem4CamNaey4kaSIae83SdCMaem4Cam3aaWbaaSqabeaacqaIYaGmaaGccqGGSaalaaa@4FD9@

where *p*_*i *_is the probability that a cell will provide *i *offspring in the next generation and *s *is a dummy variable. That is, a cell divides with probability *p*_2 _= *γ *to produce two cells; survives without dividing (that is, provides one 'offspring') with probability *p*_1 _= *δ*; and dies with probability *p*_0 _= 1 - *γ *- *δ*. The pgf enumerates all the possible outcomes after one timestep, and this is contained in the property *f *(1) = 1, or ∑*p*_*i *_= 1.

Let *Z*_*t *_be a random variable representing the total number of cells alive after *t *timesteps starting from a single cell at time 0. The moments of the probability distribution of *Z*_*t *_can be calculated from the pgf -

E(Z1|Z0=1)=∑ipi=dfds|s=1
 MathType@MTEF@5@5@+=feaafiart1ev1aaatCvAUfKttLearuWrP9MDH5MBPbIqV92AaeXatLxBI9gBaebbnrfifHhDYfgasaacH8akY=wiFfYdH8Gipec8Eeeu0xXdbba9frFj0=OqFfea0dXdd9vqai=hGuQ8kuc9pgc9s8qqaq=dirpe0xb9q8qiLsFr0=vr0=vr0dc8meaabaqaciaacaGaaeqabaqabeGadaaakeaadaabcaqaaiabdweafjabcIcaOiabdQfaAnaaBaaaleaacqaIXaqmaeqaaOGaeiiFaWNaemOwaO1aaSbaaSqaaiabicdaWaqabaGccqGH9aqpcqaIXaqmcqGGPaqkcqGH9aqpdaaeabqaaiabdMgaPjabdchaWnaaBaaaleaacqWGPbqAaeqaaaqabeqaniabggHiLdGccqGH9aqpdaWcaaqaaiabbsgaKjabdAgaMbqaaiabbsgaKjabdohaZbaaaiaawIa7amaaBaaaleaacqWGZbWCcqGH9aqpcqaIXaqmaeqaaaaa@4AAE@

and

var⁡(Zi)=∑i2pi−(∑ipi)2
 MathType@MTEF@5@5@+=feaafiart1ev1aaatCvAUfKttLearuWrP9MDH5MBPbIqV92AaeXatLxBI9gBaebbnrfifHhDYfgasaacH8akY=wiFfYdH8Gipec8Eeeu0xXdbba9frFj0=OqFfea0dXdd9vqai=hGuQ8kuc9pgc9s8qqaq=dirpe0xb9q8qiLsFr0=vr0=vr0dc8meaabaqaciaacaGaaeqabaqabeGadaaakeaacyGG2bGDcqGGHbqycqGGYbGCcqGGOaakcqWGAbGwdaWgaaWcbaGaemyAaKgabeaakiabcMcaPiabg2da9maaqaeabaGaemyAaK2aaWbaaSqabeaacqaIYaGmaaGccqWGWbaCdaWgaaWcbaGaemyAaKgabeaakiabgkHiTmaabmaabaWaaabqaeaacqWGPbqAcqWGWbaCdaWgaaWcbaGaemyAaKgabeaaaeqabeqdcqGHris5aaGccaGLOaGaayzkaaWaaWbaaSqabeaacqaIYaGmaaaabeqab0GaeyyeIuoaaaa@47D8@

=d2fds2+dfds(1−dfds)|s=1
 MathType@MTEF@5@5@+=feaafiart1ev1aaatCvAUfKttLearuWrP9MDH5MBPbIqV92AaeXatLxBI9gBaebbnrfifHhDYfgasaacH8akY=wiFfYdH8Gipec8Eeeu0xXdbba9frFj0=OqFfea0dXdd9vqai=hGuQ8kuc9pgc9s8qqaq=dirpe0xb9q8qiLsFr0=vr0=vr0dc8meaabaqaciaacaGaaeqabaqabeGadaaakeaadaabcaqaaiabg2da9maalaaabaGaeeizaq2aaWbaaSqabeaacqaIYaGmaaGccqWGMbGzaeaacqqGKbazcqWGZbWCdaahaaWcbeqaaiabikdaYaaaaaGccqGHRaWkdaWcaaqaaiabbsgaKjabdAgaMbqaaiabbsgaKjabdohaZbaadaqadaqaaiabigdaXiabgkHiTmaalaaabaGaeeizaqMaemOzaygabaGaeeizaqMaem4CamhaaaGaayjkaiaawMcaaaGaayjcSdWaaSbaaSqaaiabdohaZjabg2da9iabigdaXaqabaaaaa@49C9@

Higher moments follow in a similar way, with higher-order derivatives of the pgf. After *t *timesteps, it is straightforward to show that the expected cell counts are obtained by iterating the pgf *t *times [23]:

E(Zt|Z0=1)=df(t)ds|s=1,
 MathType@MTEF@5@5@+=feaafiart1ev1aaatCvAUfKttLearuWrP9MDH5MBPbIqV92AaeXatLxBI9gBaebbnrfifHhDYfgasaacH8akY=wiFfYdH8Gipec8Eeeu0xXdbba9frFj0=OqFfea0dXdd9vqai=hGuQ8kuc9pgc9s8qqaq=dirpe0xb9q8qiLsFr0=vr0=vr0dc8meaabaqaciaacaGaaeqabaqabeGadaaakeaadaabcaqaaiabdweafjabcIcaOiabdQfaAnaaBaaaleaacqWG0baDaeqaaOGaeiiFaWNaemOwaO1aaSbaaSqaaiabicdaWaqabaGccqGH9aqpcqaIXaqmcqGGPaqkcqGH9aqpdaWcaaqaaiabbsgaKjabdAgaMnaaCaaaleqabaGaeiikaGIaemiDaqNaeiykaKcaaaGcbaGaeeizaqMaem4CamhaaaGaayjcSdWaaSbaaSqaaiabdohaZjabg2da9iabigdaXaqabaGccqGGSaalaaa@480F@

with a similar expression to eqn. (10) for the variance, and where *f*^(*t*) ^(*s*) is *f *iterated *t *times (that is, *f*^(*t*) ^(*s*) = *f *(*f*^(*t *- 1) ^(*s*)) = *f *(*f *(*f*^(*t *- 2) ^(*s*))), *etc*.)

This models the total number of cells in the population. To keep track of the numbers of cells in each division we need to extend this procedure to a multi-type branching process in which a cell's 'type' or 'generation' is the number of divisions it has undergone, with undivided cells in generation 0. To calculate the probability distribution of cells in each generation after *t *timesteps requires a pgf that accounts for the type-label now associated with each cell and the probabilities of transition between types or generations. To do this, the pgf and the dummy variable *s *become vector-valued quantities with number of components equal to the number of cell types – in our case, the number of divisions we wish to follow using CFSE. In addition, this allows us to specify different probabilities of division and death for cells in different generations.

Define a pgf ***f ***(***s***), where ***s ***= (*s*_0_, *s*_1_, ..., *s*_*n*_) is a vector of dummy variables and ***f ***(**1**) = **1**, where **1 **is the *n *+ 1 component vector (1, 1, ..., 1). By analogy with eqn. (8), the *i*th component of ***f ***details the events that can occur to one cell in generation *i *in one timestep; namely, remain in that generation with probability *δ*_*i*_; divide to give two cells in generation *i *+ 1 with probability *γ*_*i*_; or die with probability 1 - *δ*_*i *_- *γ*_*i*_. By analogy with eqn. (11), this pgf satisfies the following; the quantity *∂f*_*i*_/*∂s*_*j *_evaluated at ***s ***= **1 **gives the expected number of offspring in generation *j *from one cell in generation *i*, after one timestep. That is,

f(s)=((1−δ0−γ0)+δ0s0+γ0s12(1−δ1−γ1)+δ1s1+γ1s22⋮δnsn+1−δn)
 MathType@MTEF@5@5@+=feaafiart1ev1aaatCvAUfKttLearuWrP9MDH5MBPbIqV92AaeXatLxBI9gBaebbnrfifHhDYfgasaacH8akY=wiFfYdH8Gipec8Eeeu0xXdbba9frFj0=OqFfea0dXdd9vqai=hGuQ8kuc9pgc9s8qqaq=dirpe0xb9q8qiLsFr0=vr0=vr0dc8meaabaqaciaacaGaaeqabaqabeGadaaakeaaieWacqWFMbGzcqGGOaakcqWFZbWCcqGGPaqkcqGH9aqpdaqadaqaauaabeqaeeaaaaqaaiabcIcaOiabigdaXiabgkHiTGGaciab+r7aKnaaBaaaleaacqaIWaamaeqaaOGaeyOeI0Iae43SdC2aaSbaaSqaaiabicdaWaqabaGccqGGPaqkcqGHRaWkcqGF0oazdaWgaaWcbaGaeGimaadabeaakiabdohaZnaaBaaaleaacqaIWaamaeqaaOGaey4kaSIae43SdC2aaSbaaSqaaiabicdaWaqabaGccqWGZbWCdaqhaaWcbaGaeGymaedabaGaeGOmaidaaaGcbaGaeiikaGIaeGymaeJaeyOeI0Iae4hTdq2aaSbaaSqaaiabigdaXaqabaGccqGHsislcqGFZoWzdaWgaaWcbaGaeGymaedabeaakiabcMcaPiabgUcaRiab+r7aKnaaBaaaleaacqaIXaqmaeqaaOGaem4Cam3aaSbaaSqaaiabigdaXaqabaGccqGHRaWkcqGFZoWzdaWgaaWcbaGaeGymaedabeaakiabdohaZnaaDaaaleaacqaIYaGmaeaacqaIYaGmaaaakeaacqWIUlstaeaacqGF0oazdaWgaaWcbaGaemOBa4gabeaakiabdohaZnaaBaaaleaacqWGUbGBaeqaaOGaey4kaSIaeGymaeJaeyOeI0Iae4hTdq2aaSbaaSqaaiabd6gaUbqabaaaaaGccaGLOaGaayzkaaaaaa@70DF@

For example, taking the first entry in ***f***, *f*_0_, in one timestep a cell in generation 0 produces an expected number of cells in generation 1 of *∂f*_0_/*∂s*_1 _= 2 *γ*_0_, and an expected number of cells in generation 0 of *∂f*_0_/*∂s*_0 _= *δ*_0 _(all derivatives evaluated at ***s ***= **1**). Notice that we assume that cells in generation *n *simply die or divide further with probability 1 - *δ*_*n*_. This would correspond, for example, to cells dividing beyond the range of generations of experimental interest, or to their CFSE fluorescence intensity becoming so low that they become indistiguishable from the CFSE-negative population in the culture – typically after 8 or 9 divisions.

Given an initial state of one cell in generation *i*, **Z**_**0 **_= **e**_***i ***_= (0, ..., 1, ..., 0), the expectation values of the cell counts after one timestep are given by

E(Z1j|Z0=ei)=∂fi∂sj|s=1≡Mij,
 MathType@MTEF@5@5@+=feaafiart1ev1aaatCvAUfKttLearuWrP9MDH5MBPbIqV92AaeXatLxBI9gBaebbnrfifHhDYfgasaacH8akY=wiFfYdH8Gipec8Eeeu0xXdbba9frFj0=OqFfea0dXdd9vqai=hGuQ8kuc9pgc9s8qqaq=dirpe0xb9q8qiLsFr0=vr0=vr0dc8meaabaqaciaacaGaaeqabaqabeGadaaakeaadaabcaqaaiabdweafjabcIcaOiabdQfaAnaaDaaaleaacqaIXaqmaeaacqWGQbGAaaGccqGG8baFieqacqWFAbGwdaWgaaWcbaGaeGimaadabeaakiabg2da9iab=vgaLnaaBaaaleaacqWGPbqAaeqaaOGaeiykaKIaeyypa0ZaaSaaaeaacqGHciITcqWGMbGzdaWgaaWcbaGaemyAaKgabeaaaOqaaiabgkGi2kabdohaZnaaBaaaleaacqWGQbGAaeqaaaaaaOGaayjcSdWaaSbaaSqaaiabdohaZjabg2da9iabigdaXaqabaGccqGHHjIUcqqGnbqtdaWgaaWcbaGaemyAaKMaemOAaOgabeaakiabcYcaSaaa@50B2@

where using (12)

M=(δ02γ00⋯⋯⋯0δ12γ10⋯⋯00δ22γ20⋯⋮⋮⋮⋮⋱⋮⋯⋯⋯0δn−12γn−1⋯⋯⋯00δn).
 MathType@MTEF@5@5@+=feaafiart1ev1aaatCvAUfKttLearuWrP9MDH5MBPbIqV92AaeXatLxBI9gBaebbnrfifHhDYfgasaacH8akY=wiFfYdH8Gipec8Eeeu0xXdbba9frFj0=OqFfea0dXdd9vqai=hGuQ8kuc9pgc9s8qqaq=dirpe0xb9q8qiLsFr0=vr0=vr0dc8meaabaqaciaacaGaaeqabaqabeGadaaakeaaieaacqWFnbqtcqGH9aqpdaqadaqaauaabeqagyaaaaaabaacciGae4hTdq2aaSbaaSqaaiabicdaWaqabaaakeaacqaIYaGmcqGFZoWzdaWgaaWcbaGaeGimaadabeaaaOqaaiabicdaWaqaaiabl+Uimbqaaiabl+Uimbqaaiabl+UimbqaaiabicdaWaqaaiab+r7aKnaaBaaaleaacqaIXaqmaeqaaaGcbaGaeGOmaiJae43SdC2aaSbaaSqaaiabigdaXaqabaaakeaacqaIWaamaeaacqWIVlctaeaacqWIVlctaeaacqaIWaamaeaacqaIWaamaeaacqGF0oazdaWgaaWcbaGaeGOmaidabeaaaOqaaiabikdaYiab+n7aNnaaBaaaleaacqaIYaGmaeqaaaGcbaGaeGimaadabaGaeS47IWeabaGaeSO7I0eabaGaeSO7I0eabaGaeSO7I0eabaGaeSO7I0eabaGaeSy8I8eabaGaeSO7I0eabaGaeS47IWeabaGaeS47IWeabaGaeS47IWeabaGaeGimaadabaGae4hTdq2aaSbaaSqaaiabd6gaUjabgkHiTiabigdaXaqabaaakeaacqaIYaGmcqGFZoWzdaWgaaWcbaGaemOBa4MaeyOeI0IaeGymaedabeaaaOqaaiabl+Uimbqaaiabl+Uimbqaaiabl+UimbqaaiabicdaWaqaaiabicdaWaqaaiab+r7aKnaaBaaaleaacqWGUbGBaeqaaaaaaOGaayjkaiaawMcaaiabc6caUaaa@7E80@

The branching process we describe here is 'memoryless' or a discrete-time Markov process with live cells making probabilistic transitions between the *n *+ 1 possible states or generations. The matrix **M **is related to the transition matrix of this Markov process, but includes not only the transition probabilities per timestep for cells in different generations, but also the expansion in population size associated with division (transition from generation *i *to *i *+ 1). In other words, it maps the cell counts at one timestep to their expected values at the following timestep. Note that we do not include dead cells as a state here – an advantage of our approach is we do not require assays for dead cells, and so do not include them as an observable in our models.

**M **can be used straightforwardly to calculate the expected cell counts at any timestep. To illustrate, consider an initial state **Z**_0 _= (*c*_0_, *c*_1_, ... *c*_*n*_) where *c*_*i *_is the number of cells in generation *i *at the beginning of the experiment. Typically, in an experiment beginning with *N *CFSE-labeled cells, **Z**_0 _= (*N*, 0, 0, ..., 0). The expected number of cells in generation *j *after one timestep can be obtained by summing the expected numbers resulting from the branching process initiated by each cell;

E(Zij|Z0)=∑i=0nciMij,
 MathType@MTEF@5@5@+=feaafiart1ev1aaatCvAUfKttLearuWrP9MDH5MBPbIqV92AaeXatLxBI9gBaebbnrfifHhDYfgasaacH8akY=wiFfYdH8Gipec8Eeeu0xXdbba9frFj0=OqFfea0dXdd9vqai=hGuQ8kuc9pgc9s8qqaq=dirpe0xb9q8qiLsFr0=vr0=vr0dc8meaabaqaciaacaGaaeqabaqabeGadaaakeaacqWGfbqrcqGGOaakieGacqWFAbGwdaqhaaWcbaGaemyAaKgabaGaemOAaOgaaOGaeiiFaWhcbeGae4NwaO1aaSbaaSqaaiabicdaWaqabaGccqGGPaqkcqGH9aqpdaaeWbqaaiabdogaJnaaBaaaleaacqWGPbqAaeqaaOGaeeyta00aaSbaaSqaaiabdMgaPjabdQgaQbqabaaabaGaemyAaKMaeyypa0JaeGimaadabaGaemOBa4ganiabggHiLdGccqGGSaalaaa@474A@

or in more compact (matrix multiplication) notation

*E *(**Z**_1_/**Z**_0_) = **Z**_0 _**M**.

After *t *timesteps, the expectation values and higher moments of the cell counts in each generation can be calculated from the pgf ***f***^(*t*) ^(***s***) (eqn. (12)) using the recursive definition [23]

***f***^(*t*) ^(**s**) = ***f ***(***f ***^(*t *- 1) ^(**s**))

As a consistency check, each component of the pgf at time *t *must satisfy the property fi(t)
 MathType@MTEF@5@5@+=feaafiart1ev1aaatCvAUfKttLearuWrP9MDH5MBPbIqV92AaeXatLxBI9gBaebbnrfifHhDYfgasaacH8akY=wiFfYdH8Gipec8Eeeu0xXdbba9frFj0=OqFfea0dXdd9vqai=hGuQ8kuc9pgc9s8qqaq=dirpe0xb9q8qiLsFr0=vr0=vr0dc8meaabaqaciaacaGaaeqabaqabeGadaaakeaacqWGMbGzdaqhaaWcbaGaemyAaKgabaGaeiikaGIaemiDaqNaeiykaKcaaaaa@32AC@ (***s ***= **1**) = 1. Since ***f ***(**1**) = **1 **from the definition (12), it follows from (14) that ***f***^(*t*) ^(**1**) = **1 **as required.

This definition of ***f***^(*t*) ^allows repeated application of the chain rule to calculate the expectation values of cell counts after *t *timesteps given any starting state **Z**_0_. For example, after two timesteps,

E(Z2j|Z0=ei)=∂fi(2)(s)∂sj|s=1=∂∂sjfj(f(s))|s=1=∑k∂fi∂sk∂fk∂sj|s=1=∑kMikMkj=(M2)ij
 MathType@MTEF@5@5@+=feaafiart1ev1aaatCvAUfKttLearuWrP9MDH5MBPbIqV92AaeXatLxBI9gBaebbnrfifHhDYfgasaacH8akY=wiFfYdH8Gipec8Eeeu0xXdbba9frFj0=OqFfea0dXdd9vqai=hGuQ8kuc9pgc9s8qqaq=dirpe0xb9q8qiLsFr0=vr0=vr0dc8meaabaqaciaacaGaaeqabaqabeGadaaakeaafaqabeqbdaaaaeaacqWGfbqrcqGGOaakcqWGAbGwdaqhaaWcbaGaeGOmaidabaGaemOAaOgaaOGaeiiFaWhcbeGae8NwaO1aaSbaaSqaaiabicdaWaqabaGccqGH9aqpcqWFLbqzdaWgaaWcbaGaemyAaKgabeaakiabcMcaPaqaaiabg2da9aqaamaaeiaabaWaaSaaaeaacqGHciITcqWGMbGzdaqhaaWcbaGaemyAaKgabaGaeiikaGIaeGOmaiJaeiykaKcaaOGaeiikaGIaem4CamNaeiykaKcabaGaeyOaIyRaem4Cam3aaSbaaSqaaiabdQgaQbqabaaaaaGccaGLiWoadaWgaaWcbaGaem4CamNaeyypa0JaeGymaedabeaaaOqaaaqaaiabg2da9aqaamaaeiaabaWaaSaaaeaacqGHciITaeaacqGHciITcqWGZbWCdaWgaaWcbaGaemOAaOgabeaaaaGccqWGMbGzdaWgaaWcbaGaemOAaOgabeaakiabcIcaOGqadiab+zgaMjabcIcaOiab+nhaZjabcMcaPiabcMcaPaGaayjcSdWaaSbaaSqaaiabdohaZjabg2da9iabigdaXaqabaaakeaaaeaacqGH9aqpaeaadaabcaqaamaaqafabaWaaSaaaeaacqGHciITcqWGMbGzdaWgaaWcbaGaemyAaKgabeaaaOqaaiabgkGi2kabdohaZnaaBaaaleaacqWGRbWAaeqaaaaakmaalaaabaGaeyOaIyRaemOzay2aaSbaaSqaaiabdUgaRbqabaaakeaacqGHciITcqWGZbWCdaWgaaWcbaGaemOAaOgabeaaaaaabaGaem4AaSgabeqdcqGHris5aaGccaGLiWoadaWgaaWcbaGaem4CamNaeyypa0JaeGymaedabeaaaOqaaaqaaiabg2da9aqaamaaqafabaGaemyta00aaSbaaSqaaiabdMgaPjabdUgaRbqabaGccqWGnbqtdaWgaaWcbaGaem4AaSMaemOAaOgabeaaaeaacqWGRbWAaeqaniabggHiLdaakeaaaeaacqGH9aqpaeaacqGGOaakcqWFnbqtdaahaaWcbeqaaiabikdaYaaakiabcMcaPmaaBaaaleaacqWGPbqAcqWGQbGAaeqaaaaaaaa@94CD@

By simple extension, expected cell counts at later timepoints can be calculated with repeated matrix multiplication using **M **-

*E *(**Z**_*t*_/**Z**_0_) = **Z**_**0 **_**M**^*t*^.

The covariances of cell counts in each generation, and higher moments, can be calculated in a similar way. Our method requires the first two moments, and so we wish to calculate **V**_*t*_, the covariance matrix of cell counts in each generation after *t *timesteps given initial cell counts **Z**_0_, or

cov⁡(Zti,Ztj)≡(Vt)ij=E(ZtiZtj)−E(Zti)E(Ztj).
 MathType@MTEF@5@5@+=feaafiart1ev1aaatCvAUfKttLearuWrP9MDH5MBPbIqV92AaeXatLxBI9gBaebbnrfifHhDYfgasaacH8akY=wiFfYdH8Gipec8Eeeu0xXdbba9frFj0=OqFfea0dXdd9vqai=hGuQ8kuc9pgc9s8qqaq=dirpe0xb9q8qiLsFr0=vr0=vr0dc8meaabaqaciaacaGaaeqabaqabeGadaaakeaacyGGJbWycqGGVbWBcqGG2bGDcqGGOaakcqWGAbGwdaqhaaWcbaGaemiDaqhabaGaemyAaKgaaOGaeiilaWIaemOwaO1aa0baaSqaaiabdsha0bqaaiabdQgaQbaakiabcMcaPiabggMi6kabcIcaOGqabiab=zfawnaaBaaaleaacqWG0baDaeqaaOGaeiykaKYaaSbaaSqaaiabdMgaPjabdQgaQbqabaGccqGH9aqpcqWGfbqrcqGGOaakcqWGAbGwdaqhaaWcbaGaemiDaqhabaGaemyAaKgaaOGaemOwaO1aa0baaSqaaiabdsha0bqaaiabdQgaQbaakiabcMcaPiabgkHiTiabdweafjabcIcaOiabdQfaAnaaDaaaleaacqWG0baDaeaacqWGPbqAaaGccqGGPaqkcqWGfbqrcqGGOaakcqWGAbGwdaqhaaWcbaGaemiDaqhabaGaemOAaOgaaOGaeiykaKIaeiOla4caaa@615F@

As illustrated in eqn. (10), this can be calculated from derivatives of the pgf. For instance, given one cell in generation *k *(that is, **Z**_0 _= **e**_*k*_), after one timestep

cov⁡(Z1i,Z1j)=∂2fk∂si∂sj−∂fk∂si∂fk∂sj|s=1
 MathType@MTEF@5@5@+=feaafiart1ev1aaatCvAUfKttLearuWrP9MDH5MBPbIqV92AaeXatLxBI9gBaebbnrfifHhDYfgasaacH8akY=wiFfYdH8Gipec8Eeeu0xXdbba9frFj0=OqFfea0dXdd9vqai=hGuQ8kuc9pgc9s8qqaq=dirpe0xb9q8qiLsFr0=vr0=vr0dc8meaabaqaciaacaGaaeqabaqabeGadaaakeaadaabcaqaaiGbcogaJjabc+gaVjabcAha2jabcIcaOiabdQfaAnaaDaaaleaacqaIXaqmaeaacqWGPbqAaaGccqGGSaalcqWGAbGwdaqhaaWcbaGaeGymaedabaGaemOAaOgaaOGaeiykaKIaeyypa0ZaaSaaaeaacqGHciITdaahaaWcbeqaaiabikdaYaaakiabdAgaMnaaBaaaleaacqWGRbWAaeqaaaGcbaGaeyOaIyRaem4Cam3aaSbaaSqaaiabdMgaPbqabaGccqGHciITcqWGZbWCdaWgaaWcbaGaemOAaOgabeaaaaGccqGHsisldaWcaaqaaiabgkGi2kabdAgaMnaaBaaaleaacqWGRbWAaeqaaaGcbaGaeyOaIyRaem4Cam3aaSbaaSqaaiabdMgaPbqabaaaaOWaaSaaaeaacqGHciITcqWGMbGzdaWgaaWcbaGaem4AaSgabeaaaOqaaiabgkGi2kabdohaZnaaBaaaleaacqWGQbGAaeqaaaaaaOGaayjcSdWaaSbaaSqaaiabdohaZjabg2da9iabigdaXaqabaaaaa@61E7@

and

var⁡(Z1i)=∂2fk∂si2+∂fk∂si(1−∂fk∂si)|s=1.
 MathType@MTEF@5@5@+=feaafiart1ev1aaatCvAUfKttLearuWrP9MDH5MBPbIqV92AaeXatLxBI9gBaebbnrfifHhDYfgasaacH8akY=wiFfYdH8Gipec8Eeeu0xXdbba9frFj0=OqFfea0dXdd9vqai=hGuQ8kuc9pgc9s8qqaq=dirpe0xb9q8qiLsFr0=vr0=vr0dc8meaabaqaciaacaGaaeqabaqabeGadaaakeaadaabcaqaaiGbcAha2jabcggaHjabckhaYjabcIcaOiabdQfaAnaaDaaaleaacqaIXaqmaeaacqWGPbqAaaGccqGGPaqkcqGH9aqpdaWcaaqaaiabgkGi2oaaCaaaleqabaGaeGOmaidaaOGaemOzay2aaSbaaSqaaiabdUgaRbqabaaakeaacqGHciITcqWGZbWCdaqhaaWcbaGaemyAaKgabaGaeGOmaidaaaaakiabgUcaRmaalaaabaGaeyOaIyRaemOzay2aaSbaaSqaaiabdUgaRbqabaaakeaacqGHciITcqWGZbWCdaWgaaWcbaGaemyAaKgabeaaaaGcdaqadaqaaiabigdaXiabgkHiTmaalaaabaGaeyOaIyRaemOzay2aaSbaaSqaaiabdUgaRbqabaaakeaacqGHciITcqWGZbWCdaWgaaWcbaGaemyAaKgabeaaaaaakiaawIcacaGLPaaaaiaawIa7amaaBaaaleaacqWGZbWCcqGH9aqpcqaIXaqmaeqaaOGaeiOla4caaa@5E1A@

At later timepoints these quantities can be calculated, again using the recursive definition of the pgf (eqn. (14)) [23]

Vt+1=MTVtM+∑k=0nvkE(Ztk),
 MathType@MTEF@5@5@+=feaafiart1ev1aaatCvAUfKttLearuWrP9MDH5MBPbIqV92AaeXatLxBI9gBaebbnrfifHhDYfgasaacH8akY=wiFfYdH8Gipec8Eeeu0xXdbba9frFj0=OqFfea0dXdd9vqai=hGuQ8kuc9pgc9s8qqaq=dirpe0xb9q8qiLsFr0=vr0=vr0dc8meaabaqaciaacaGaaeqabaqabeGadaaakeaaieqacqWFwbGvdaWgaaWcbaGaemiDaqNaey4kaSIaeGymaedabeaakiabg2da9iab=1eannaaCaaaleqabaGaemivaqfaaOGae8Nvay1aaSbaaSqaaiabdsha0bqabaGccqWFnbqtcqGHRaWkdaaeWbqaaiab=zha2naaBaaaleaacqWGRbWAaeqaaOGaemyrauKaeiikaGIaemOwaO1aa0baaSqaaiabdsha0bqaaiabdUgaRbaakiabcMcaPaWcbaGaem4AaSMaeyypa0JaeGimaadabaGaemOBa4ganiabggHiLdGccqGGSaalaaa@4BB9@

where **M**^*T *^is the transpose of **M **and the *n *+ 1 matrices **v**_*k *_are the covariance matrices for one timestep for one cell beginning in state **Z**_*k *_= **e**_*k*_, calculated from the pgf fk(1)
 MathType@MTEF@5@5@+=feaafiart1ev1aaatCvAUfKttLearuWrP9MDH5MBPbIqV92AaeXatLxBI9gBaebbnrfifHhDYfgasaacH8akY=wiFfYdH8Gipec8Eeeu0xXdbba9frFj0=OqFfea0dXdd9vqai=hGuQ8kuc9pgc9s8qqaq=dirpe0xb9q8qiLsFr0=vr0=vr0dc8meaabaqaciaacaGaaeqabaqabeGadaaakeaacqWGMbGzdaqhaaWcbaGaem4AaSgabaGaeiikaGIaeGymaeJaeiykaKcaaaaa@322F@ – that is, the off-diagonal elements of **v**_*k *_are given by eqn. (16), and the diagonal elements by eqn. (17). For example,

v0=(δ0(1−δ0)−2γ0δ00⋯−2γ0δ04γ0(1−γ0)0000⋯⋮⋮⋮⋱)
 MathType@MTEF@5@5@+=feaafiart1ev1aaatCvAUfKttLearuWrP9MDH5MBPbIqV92AaeXatLxBI9gBaebbnrfifHhDYfgasaacH8akY=wiFfYdH8Gipec8Eeeu0xXdbba9frFj0=OqFfea0dXdd9vqai=hGuQ8kuc9pgc9s8qqaq=dirpe0xb9q8qiLsFr0=vr0=vr0dc8meaabaqaciaacaGaaeqabaqabeGadaaakeaaieqacqWF2bGDdaWgaaWcbaGaeGimaadabeaakiabg2da9maabmaabaqbaeqabqabaaaaaeaaiiGacqGF0oazdaWgaaWcbaGaeGimaadabeaakiabcIcaOiabigdaXiabgkHiTiab+r7aKnaaBaaaleaacqaIWaamaeqaaOGaeiykaKcabaGaeyOeI0IaeGOmaiJae43SdC2aaSbaaSqaaiabicdaWaqabaGccqGF0oazdaWgaaWcbaGaeGimaadabeaaaOqaaiabicdaWaqaaiabl+UimbqaaiabgkHiTiabikdaYiab+n7aNnaaBaaaleaacqaIWaamaeqaaOGae4hTdq2aaSbaaSqaaiabicdaWaqabaaakeaacqaI0aancqGFZoWzdaWgaaWcbaGaeGimaadabeaakiabcIcaOiabigdaXiabgkHiTiab+n7aNnaaBaaaleaacqaIWaamaeqaaOGaeiykaKcabaGaeGimaadabaaabaGaeGimaadabaGaeGimaadabaGaeGimaadabaGaeS47IWeabaGaeSO7I0eabaGaeSO7I0eabaGaeSO7I0eabaGaeSy8I8eaaaGaayjkaiaawMcaaaaa@6437@

For a general initial state **Z**_0 _= (*c*_0_, *c*_1_, ..., *c*_*n*_), the assumption of independence of the branching processes initiated by each cell gives **V**_1 _= ∑_*i*_*c*_*i*_**v**_*i*_. Again, a typical CFSE experiment might start with *N *cells in generation 0, yielding **V**_1 _= *N ***v**_0_.

This framework makes it straightforward to include time-varying probabilities of division and quiescence – that is, *γ*_*i *_(*t*) and *δ*_*i *_(*t*). Essentially, the pgf and hence the matrices **M **and **v**_*i *_become time dependent. Let **M**_*t *_be the transition matrix that maps cell counts at timestep *t *to their expected values at time *t *+ 1, as in eqn. (13) but now using the parameters *γ*_*i *_(*t*) and *δ*_*i *_(*t*); and let **v**_*i, t *_be the covariance matrix of the cell counts generated in one timestep by a single cell in generation *i *at time *t*. Equations (15) and (18) then become

E(Zt|Z0)=Z0∏j=0t−1Mj,Vt+1=MtTVtMt+∑k=0nvk,tE(Ztk).
 MathType@MTEF@5@5@+=feaafiart1ev1aaatCvAUfKttLearuWrP9MDH5MBPbIqV92AaeXatLxBI9gBaebbnrfifHhDYfgasaacH8akY=wiFfYdH8Gipec8Eeeu0xXdbba9frFj0=OqFfea0dXdd9vqai=hGuQ8kuc9pgc9s8qqaq=dirpe0xb9q8qiLsFr0=vr0=vr0dc8meaabaqaciaacaGaaeqabaqabeGadaaakeaafaqabeGabaaabaGaemyrauKaeiikaGccbeGae8NwaO1aaSbaaSqaaiabdsha0bqabaGccqGG8baFcqWFAbGwdaWgaaWcbaGaeGimaadabeaakiabcMcaPiabg2da9iab=PfaAnaaBaaaleaacqaIWaamaeqaaOWaaebCaeaacqWFnbqtdaWgaaWcbaGaemOAaOgabeaaaeaacqWGQbGAcqGH9aqpcqaIWaamaeaacqWG0baDcqGHsislcqaIXaqma0Gaey4dIunakiabcYcaSaqaaiab=zfawnaaBaaaleaacqWG0baDcqGHRaWkcqaIXaqmaeqaaOGaeyypa0Jae8xta00aa0baaSqaaiabdsha0bqaaiabdsfaubaakiab=zfawnaaBaaaleaacqWG0baDaeqaaOGae8xta00aaSbaaSqaaiabdsha0bqabaGccqGHRaWkdaaeWbqaaiab=zha2naaBaaaleaacqWGRbWAcqGGSaalcqWG0baDaeqaaOGaemyrauKaeiikaGIaemOwaO1aa0baaSqaaiabdsha0bqaaiabdUgaRbaakiabcMcaPaWcbaGaem4AaSMaeyypa0JaeGimaadabaGaemOBa4ganiabggHiLdGccqGGUaGlaaaaaa@6A6F@

## Authors' contributions

AY and CC contributed equally to this study. They jointly developed and implemented the discrete time formalism and QL estimation procedure, performed the analysis of all datasets, and co-wrote the manuscript. J. Stark performed preliminary calculations of the covariance structure, suggested the QL approach and provided technical advice. J. Strid performed the T cell proliferation assay. SM was involved in the early conception of the idea of using branching processes and performed and described the analysis of the continuous-time case. J. Stark, AG and RC conceived the study and provided substantial input to the manuscript.

## Appendices

### 1. The Continuous-Time Analogue

The continuous-time analogue of a Galton-Watson process is the Markov age dependent process. This is characterised by cells having life spans that are exponentially distributed random variables with parameter *λ *[21]. This is conceptually a quite different model of cell behaviour to that described above. It can be compared to a limit of the Smith-Martin model in which death can only occur during the B phase (during which cells are actively dividing) and the duration of this phase approaches zero. Thus, for example, it may be a reasonable model for slow homeostatic division in which the average time spent in the cell cycle is negligible compared to the time spent in quiescence.

For a single type this process is defined by the partial differential equation:

∂F(s,t)∂t=−λ(F(s,t)−f(F(s,t)))
 MathType@MTEF@5@5@+=feaafiart1ev1aaatCvAUfKttLearuWrP9MDH5MBPbIqV92AaeXatLxBI9gBaebbnrfifHhDYfgasaacH8akY=wiFfYdH8Gipec8Eeeu0xXdbba9frFj0=OqFfea0dXdd9vqai=hGuQ8kuc9pgc9s8qqaq=dirpe0xb9q8qiLsFr0=vr0=vr0dc8meaabaqaciaacaGaaeqabaqabeGadaaakeaadaWcaaqaaiabgkGi2kabdAeagjabcIcaOiabdohaZjabcYcaSiabdsha0jabcMcaPaqaaiabgkGi2kabdsha0baacqGH9aqpcqGHsisliiGacqWF7oaBcqGGOaakcqWGgbGrcqGGOaakcqWGZbWCcqGGSaalcqWG0baDcqGGPaqkcqGHsislcqWGMbGzcqGGOaakcqWGgbGrcqGGOaakcqWGZbWCcqGGSaalcqWG0baDcqGGPaqkcqGGPaqkcqGGPaqkaaa@4DE2@

with initial condition *F *(*s*, 0) = *s*. Here, *F *(*s, t*) is the 'process' pgf, derivatives of which generate the moments of the distribution of the total cell numbers at time *t*. For example,

E(Z(t))=ddsF(s,t)|s=1.
 MathType@MTEF@5@5@+=feaafiart1ev1aaatCvAUfKttLearuWrP9MDH5MBPbIqV92AaeXatLxBI9gBaebbnrfifHhDYfgasaacH8akY=wiFfYdH8Gipec8Eeeu0xXdbba9frFj0=OqFfea0dXdd9vqai=hGuQ8kuc9pgc9s8qqaq=dirpe0xb9q8qiLsFr0=vr0=vr0dc8meaabaqaciaacaGaaeqabaqabeGadaaakeaadaabcaqaaiabdweafjabcIcaOiabdQfaAjabcIcaOiabdsha0jabcMcaPiabcMcaPiabg2da9maalaaabaGaeeizaqgabaGaeeizaqMaem4CamhaaiabdAeagjabcIcaOiabdohaZjabcYcaSiabdsha0jabcMcaPaGaayjcSdWaaSbaaSqaaiabdohaZjabg2da9iabigdaXaqabaGccqGGUaGlaaa@4590@

The quantity *f *(*s*) is a progeny pgf which dictates the distribution of offspring numbers. This can be generalized to the multitype case where cells that have divided *k *times are assigned a type-label *k*, where *k *= 0, 1, ..., *η *and *η *is the highest generation number of interest or observable. Denoting **s **as the vector of dummy variables *s*_*k *_*i.e*. **s **= (*s*_0_, *s*_1_, ..., *s*_*η*_), each parental type *k *produces offspring according to the progeny pgf *f*_*k *_(**s**). Here, a process started by a cell of type *k *is described by a process pgf *F*_*k *_(*s, t*) where the lifetime of each individual of type *k *is exponentially distributed with parameter *λ*_*k*_. Denoting ***F***, ***f ***and ***λ ***as vectors containing the process and progeny pgfs in addition to the *λ*_*k *_for each type respectively, we obtain a system of partial differential equations for a multitype continuous-time branching process represented by the general equation

∂Fk(s,t)∂t=−λk(Fk(s,t)−fk(F(s,t)))
 MathType@MTEF@5@5@+=feaafiart1ev1aaatCvAUfKttLearuWrP9MDH5MBPbIqV92AaeXatLxBI9gBaebbnrfifHhDYfgasaacH8akY=wiFfYdH8Gipec8Eeeu0xXdbba9frFj0=OqFfea0dXdd9vqai=hGuQ8kuc9pgc9s8qqaq=dirpe0xb9q8qiLsFr0=vr0=vr0dc8meaabaqaciaacaGaaeqabaqabeGadaaakeaadaWcaaqaaiabgkGi2kabdAeagnaaBaaaleaacqWGRbWAaeqaaOGaeiikaGccbmGae83CamNaeiilaWIaemiDaqNaeiykaKcabaGaeyOaIyRaemiDaqhaaiabg2da9iabgkHiTGGaciab+T7aSnaaBaaaleaacqWGRbWAaeqaaOGaeiikaGIaemOray0aaSbaaSqaaiabdUgaRbqabaGccqGGOaakcqWFZbWCcqGGSaalcqWG0baDcqGGPaqkcqGHsislcqWGMbGzdaWgaaWcbaGaem4AaSgabeaakiabcIcaOiab=zeagjabcIcaOiab=nhaZjabcYcaSiabdsha0jabcMcaPiabcMcaPiabcMcaPaaa@5431@

with initial conditions *F*_*k *_(***s***, 0) = *s*_*k*_. We now demonstrate the calculation of the expected number of cells and the covariance matrix for such a process, where each type corresponds to a generation. We show the simplest example in which all generations have identically distributed lifetimes, i.e. *λ*_*k *_= *λ*. At the end of its lifetime a cell either divides or dies with probabilities *γ *or *α *= 1 - *γ *respectively. We also set the maximum number of types *η *to be one greater than the maximum number of divisions we wish to model. Solution of our system is simplified by modeling the normalized cell counts; the cell count for each generation *k *is multiplied by 2^-*k*^. This can be interpreted as following the probabilistic evolution of CFSE dye from one generation to another. The progeny pgfs for each parental type are therefore *f*_*k *_(***s***) = *α *+ *γ s*_*k*+1 _for *k *<*η *and *f*_*η *_(***s***) = *α *+ *γ s*_*η *_for *k *= *η*. Denoting *F*_*k *_= *F*_*k *_(***s**, t*) we therefore obtain the following cascade system of PDEs:

∂Fk∂t=−λFk+λ(α+γFk+1)
 MathType@MTEF@5@5@+=feaafiart1ev1aaatCvAUfKttLearuWrP9MDH5MBPbIqV92AaeXatLxBI9gBaebbnrfifHhDYfgasaacH8akY=wiFfYdH8Gipec8Eeeu0xXdbba9frFj0=OqFfea0dXdd9vqai=hGuQ8kuc9pgc9s8qqaq=dirpe0xb9q8qiLsFr0=vr0=vr0dc8meaabaqaciaacaGaaeqabaqabeGadaaakeaadaWcaaqaaiabgkGi2kabdAeagnaaBaaaleaacqWGRbWAaeqaaaGcbaGaeyOaIyRaemiDaqhaaiabg2da9iabgkHiTGGaciab=T7aSjabdAeagnaaBaaaleaacqWGRbWAaeqaaOGaey4kaSIae83UdWMaeiikaGIae8xSdeMaey4kaSIae83SdCMaemOray0aaSbaaSqaaiabdUgaRjabgUcaRiabigdaXaqabaGccqGGPaqkaaa@46D8@

for *k *<*η *and

∂Fη∂t=−λFη+λ(α+γFη)
 MathType@MTEF@5@5@+=feaafiart1ev1aaatCvAUfKttLearuWrP9MDH5MBPbIqV92AaeXatLxBI9gBaebbnrfifHhDYfgasaacH8akY=wiFfYdH8Gipec8Eeeu0xXdbba9frFj0=OqFfea0dXdd9vqai=hGuQ8kuc9pgc9s8qqaq=dirpe0xb9q8qiLsFr0=vr0=vr0dc8meaabaqaciaacaGaaeqabaqabeGadaaakeaadaWcaaqaaiabgkGi2kabdAeagnaaBaaaleaaiiGacqWF3oaAaeqaaaGcbaGaeyOaIyRaemiDaqhaaiabg2da9iabgkHiTiab=T7aSjabdAeagnaaBaaaleaacqWF3oaAaeqaaOGaey4kaSIae83UdWMaeiikaGIae8xSdeMaey4kaSIae83SdCMaemOray0aaSbaaSqaaiab=D7aObqabaGccqGGPaqkaaa@45DE@

for *k *= *η*. Using the integrating factor *e*^*λt *^this system of equations can easily be solved by back substitution yielding

*F*_*η *_= 1 + *e*^-*λαt *^(*s*_*η *_- 1)

and for *k *<*η*

Fk=1+e−λαt(sη−1)+e−λt∑i=0η−k(si+k−sη)(λγt)ii!.
 MathType@MTEF@5@5@+=feaafiart1ev1aaatCvAUfKttLearuWrP9MDH5MBPbIqV92AaeXatLxBI9gBaebbnrfifHhDYfgasaacH8akY=wiFfYdH8Gipec8Eeeu0xXdbba9frFj0=OqFfea0dXdd9vqai=hGuQ8kuc9pgc9s8qqaq=dirpe0xb9q8qiLsFr0=vr0=vr0dc8meaabaqaciaacaGaaeqabaqabeGadaaakeaacqWGgbGrdaWgaaWcbaGaem4AaSgabeaakiabg2da9iabigdaXiabgUcaRiabdwgaLnaaCaaaleqabaGaeyOeI0ccciGae83UdWMae8xSdeMaemiDaqhaaOGaeiikaGIaem4Cam3aaSbaaSqaaiab=D7aObqabaGccqGHsislcqaIXaqmcqGGPaqkcqGHRaWkcqWGLbqzdaahaaWcbeqaaiabgkHiTiab=T7aSjabdsha0baakmaaqahabaGaeiikaGIaem4Cam3aaSbaaSqaaiabdMgaPjabgUcaRiabdUgaRbqabaGccqGHsislcqWGZbWCdaWgaaWcbaGae83TdGgabeaakiabcMcaPmaalaaabaGaeiikaGIae83UdWMae83SdCMaemiDaqNaeiykaKYaaWbaaSqabeaacqWGPbqAaaaakeaacqWGPbqAcqGGHaqiaaaaleaacqWGPbqAcqGH9aqpcqaIWaamaeaacqWF3oaAcqGHsislcqWGRbWAa0GaeyyeIuoakiabc6caUaaa@6698@

If we start with one undivided cell at time zero the expectation of the normalized cell count *E *(*N*_*k*_) for generation *k *is obtained by differentiating *F*_0 _with respect to *s*_*k *_and subsequently setting all *s*_*k *_= 1. In this simple case the derivatives of *F*_0 _do not include terms in *s*_*k *_and this last step can be omitted.

From (19) we therefore obtain the expected normalized cell counts

E(Nk)=e−λt(λγt)kk!.
 MathType@MTEF@5@5@+=feaafiart1ev1aaatCvAUfKttLearuWrP9MDH5MBPbIqV92AaeXatLxBI9gBaebbnrfifHhDYfgasaacH8akY=wiFfYdH8Gipec8Eeeu0xXdbba9frFj0=OqFfea0dXdd9vqai=hGuQ8kuc9pgc9s8qqaq=dirpe0xb9q8qiLsFr0=vr0=vr0dc8meaabaqaciaacaGaaeqabaqabeGadaaakeaacqWGfbqrcqGGOaakcqWGobGtdaWgaaWcbaGaem4AaSgabeaakiabcMcaPiabg2da9maalaaabaGaemyzau2aaWbaaSqabeaacqGHsisliiGacqWF7oaBcqWG0baDaaGccqGGOaakcqWF7oaBcqWFZoWzcqWG0baDcqGGPaqkdaahaaWcbeqaaiabdUgaRbaaaOqaaiabdUgaRjabcgcaHaaacqGGUaGlaaa@43FB@

We obtain the expected cell counts *E *(*Z*_*k*_) by reversing the normalization procedure, obtaining

E(Zk)=e−λt(2λγt)kk!.
 MathType@MTEF@5@5@+=feaafiart1ev1aaatCvAUfKttLearuWrP9MDH5MBPbIqV92AaeXatLxBI9gBaebbnrfifHhDYfgasaacH8akY=wiFfYdH8Gipec8Eeeu0xXdbba9frFj0=OqFfea0dXdd9vqai=hGuQ8kuc9pgc9s8qqaq=dirpe0xb9q8qiLsFr0=vr0=vr0dc8meaabaqaciaacaGaaeqabaqabeGadaaakeaacqWGfbqrcqGGOaakcqWGAbGwdaWgaaWcbaGaem4AaSgabeaakiabcMcaPiabg2da9maalaaabaGaemyzau2aaWbaaSqabeaacqGHsisliiGacqWF7oaBcqWG0baDaaGccqGGOaakcqaIYaGmcqWF7oaBcqWFZoWzcqWG0baDcqGGPaqkdaahaaWcbeqaaiabdUgaRbaaaOqaaiabdUgaRjabcgcaHaaacqGGUaGlaaa@4505@

The off-diagonal and diagonal terms of the covariance matrix of the quantities *Z*_*k *_can be obtained from eqns. (16) and (17) respectively. The second derivatives are zero since, as noted above, the first derivatives of *F*_*k *_do not contain terms in *s*_*k*_, giving

cov⁡(Zi,Zj)=−e−2λt(2λγt)i+ji!j!
 MathType@MTEF@5@5@+=feaafiart1ev1aaatCvAUfKttLearuWrP9MDH5MBPbIqV92AaeXatLxBI9gBaebbnrfifHhDYfgasaacH8akY=wiFfYdH8Gipec8Eeeu0xXdbba9frFj0=OqFfea0dXdd9vqai=hGuQ8kuc9pgc9s8qqaq=dirpe0xb9q8qiLsFr0=vr0=vr0dc8meaabaqaciaacaGaaeqabaqabeGadaaakeaacyGGJbWycqGGVbWBcqGG2bGDcqGGOaakcqWGAbGwdaWgaaWcbaGaemyAaKgabeaakiabcYcaSiabdQfaAnaaBaaaleaacqWGQbGAaeqaaOGaeiykaKIaeyypa0JaeyOeI0YaaSaaaeaacqWGLbqzdaahaaWcbeqaaiabgkHiTiabikdaYGGaciab=T7aSjabdsha0baakiabcIcaOiabikdaYiab=T7aSjab=n7aNjabdsha0jabcMcaPmaaCaaaleqabaGaemyAaKMaey4kaSIaemOAaOgaaaGcbaGaemyAaKMaeiyiaeIaemOAaOMaeiyiaecaaaaa@5121@

and

var⁡(Zi)=e−λt(2λγt)ii!−e−2λt(2λγt)2ii!2.
 MathType@MTEF@5@5@+=feaafiart1ev1aaatCvAUfKttLearuWrP9MDH5MBPbIqV92AaeXatLxBI9gBaebbnrfifHhDYfgasaacH8akY=wiFfYdH8Gipec8Eeeu0xXdbba9frFj0=OqFfea0dXdd9vqai=hGuQ8kuc9pgc9s8qqaq=dirpe0xb9q8qiLsFr0=vr0=vr0dc8meaabaqaciaacaGaaeqabaqabeGadaaakeaacyGG2bGDcqGGHbqycqGGYbGCcqGGOaakcqWGAbGwdaWgaaWcbaGaemyAaKgabeaakiabcMcaPiabg2da9maalaaabaGaemyzau2aaWbaaSqabeaacqGHsisliiGacqWF7oaBcqWG0baDaaGccqGGOaakcqaIYaGmcqWF7oaBcqWFZoWzcqWG0baDcqGGPaqkdaahaaWcbeqaaiabdMgaPbaaaOqaaiabdMgaPjabcgcaHaaacqGHsisldaWcaaqaaiabdwgaLnaaCaaaleqabaGaeyOeI0IaeGOmaiJae83UdWMaemiDaqhaaOGaeiikaGIaeGOmaiJae83UdWMae83SdCMaemiDaqNaeiykaKYaaWbaaSqabeaacqaIYaGmcqWGPbqAaaaakeaacqWGPbqAcqGGHaqidaahaaWcbeqaaiabikdaYaaaaaGccqGGUaGlaaa@5CD0@

For a two-generation model beginning with one cell in generation zero we therefore obtain the covariance matrix

v0(t)=(e−λt−e−2λt−2e−2λtθ−2e−2λtθ2eλtθ−4e−2λtθ2)
 MathType@MTEF@5@5@+=feaafiart1ev1aaatCvAUfKttLearuWrP9MDH5MBPbIqV92AaeXatLxBI9gBaebbnrfifHhDYfgasaacH8akY=wiFfYdH8Gipec8Eeeu0xXdbba9frFj0=OqFfea0dXdd9vqai=hGuQ8kuc9pgc9s8qqaq=dirpe0xb9q8qiLsFr0=vr0=vr0dc8meaabaqaciaacaGaaeqabaqabeGadaaakeaaieqacqWF2bGDdaWgaaWcbaGaeGimaadabeaakiabcIcaOiabdsha0jabcMcaPiabg2da9maabmaabaqbaeqabiGaaaqaaiabdwgaLnaaCaaaleqabaGaeyOeI0ccciGae43UdWMaemiDaqhaaOGaeyOeI0Iaemyzau2aaWbaaSqabeaacqGHsislcqaIYaGmcqGF7oaBcqWG0baDaaaakeaacqGHsislcqaIYaGmcqWGLbqzdaahaaWcbeqaaiabgkHiTiabikdaYiab+T7aSjabdsha0baakiab+H7aXbqaaiabgkHiTiabikdaYiabdwgaLnaaCaaaleqabaGaeyOeI0IaeGOmaiJae43UdWMaemiDaqhaaOGae4hUdehabaGaeGOmaiJaemyzau2aaWbaaSqabeaacqGF7oaBcqWG0baDaaGccqGF4oqCcqGHsislcqaI0aancqWGLbqzdaahaaWcbeqaaiabgkHiTiabikdaYiab+T7aSjabdsha0baakiab+H7aXnaaCaaaleqabaGaeGOmaidaaaaaaOGaayjkaiaawMcaaaaa@68E1@

where *θ *= *λ γ t*. As before, given an initial state **Z**_0 _= (*c*_0_, *c*_1_, ..., *c*_*n*_) the covariance matrix at time *t *will be **V**(*t*) = ∑_*i*_*c*_*i*_**v**_*i*_(*t*). Further extension of this model can be achieved through altering constraints on *λ *and *γ*. In the case of the probability of division *γ *this parameter can either become a function of generation *k *or a function of *t*. In the latter case the system of equations may become inhomogeneous with respect to time and therefore its solution may prove difficult. A much more general approach to continuous-time models is the use of Bellman-Harris processes where the distribution of lifetimes is not restricted to the exponential. However, many such processes are non-Markovian and so also become significantly harder to analyse.

### 2. Computing an exact likelihood

The pgf allows us in principle to write down an exact likelihood for any given set of cell counts, using combinations of its derivatives. To illustrate for a simple single-type discrete-time branching process, after *t *timesteps the pgf can be written

f(t)(s)=∑i=02tpisi
 MathType@MTEF@5@5@+=feaafiart1ev1aaatCvAUfKttLearuWrP9MDH5MBPbIqV92AaeXatLxBI9gBaebbnrfifHhDYfgasaacH8akY=wiFfYdH8Gipec8Eeeu0xXdbba9frFj0=OqFfea0dXdd9vqai=hGuQ8kuc9pgc9s8qqaq=dirpe0xb9q8qiLsFr0=vr0=vr0dc8meaabaqaciaacaGaaeqabaqabeGadaaakeaacqWGMbGzdaahaaWcbeqaaiabcIcaOiabdsha0jabcMcaPaaakiabcIcaOiabdohaZjabcMcaPiabg2da9maaqahabaGaemiCaa3aaSbaaSqaaiabdMgaPbqabaGccqWGZbWCdaahaaWcbeqaaiabdMgaPbaaaeaacqWGPbqAcqGH9aqpcqaIWaamaeaacqaIYaGmdaahaaadbeqaaiabdsha0baaa0GaeyyeIuoaaaa@438A@

and so the probability of *i *cells surviving at this time given a single cell at time 0 is

pi=1n!didsif(t)(s)|s=0.
 MathType@MTEF@5@5@+=feaafiart1ev1aaatCvAUfKttLearuWrP9MDH5MBPbIqV92AaeXatLxBI9gBaebbnrfifHhDYfgasaacH8akY=wiFfYdH8Gipec8Eeeu0xXdbba9frFj0=OqFfea0dXdd9vqai=hGuQ8kuc9pgc9s8qqaq=dirpe0xb9q8qiLsFr0=vr0=vr0dc8meaabaqaciaacaGaaeqabaqabeGadaaakeaadaabcaqaaiabdchaWnaaBaaaleaacqWGPbqAaeqaaOGaeyypa0ZaaSaaaeaacqaIXaqmaeaacqWGUbGBcqGGHaqiaaWaaSaaaeaacqqGKbazdaahaaWcbeqaaiabdMgaPbaaaOqaaiabbsgaKjabdohaZnaaCaaaleqabaGaemyAaKgaaaaakiabdAgaMnaaCaaaleqabaGaeiikaGIaemiDaqNaeiykaKcaaOGaeiikaGIaem4CamNaeiykaKcacaGLiWoadaWgaaWcbaGaem4CamNaeyypa0JaeGimaadabeaakiabc6caUaaa@48FF@

Starting with *N *cells a time 0, the probability of observing *M *cells in total after *t *timesteps is then the quantity

P(M|N)=∑(q1,...,qN)M!q1!...qN!pq1pq2...pqN,
 MathType@MTEF@5@5@+=feaafiart1ev1aaatCvAUfKttLearuWrP9MDH5MBPbIqV92AaeXatLxBI9gBaebbnrfifHhDYfgasaacH8akY=wiFfYdH8Gipec8Eeeu0xXdbba9frFj0=OqFfea0dXdd9vqai=hGuQ8kuc9pgc9s8qqaq=dirpe0xb9q8qiLsFr0=vr0=vr0dc8meaabaqaciaacaGaaeqabaqabeGadaaakeaacqWGqbaucqGGOaakcqWGnbqtcqGG8baFcqWGobGtcqGGPaqkcqGH9aqpdaaeqbqaamaalaaabaGaemyta0KaeiyiaecabaGaemyCae3aaSbaaSqaaiabigdaXaqabaGccqGGHaqicqGGUaGlcqGGUaGlcqGGUaGlcqWGXbqCdaWgaaWcbaGaemOta4eabeaakiabcgcaHaaaaSqaaiabcIcaOiabdghaXnaaBaaameaacqaIXaqmaeqaaSGaeiilaWIaeiOla4IaeiOla4IaeiOla4IaeiilaWIaemyCae3aaSbaaWqaaiabd6eaobqabaWccqGGPaqkaeqaniabggHiLdGccqWGWbaCdaWgaaWcbaGaemyCae3aaSbaaWqaaiabigdaXaqabaaaleqaaOGaemiCaa3aaSbaaSqaaiabdghaXnaaBaaameaacqaIYaGmaeqaaaWcbeaakiabc6caUiabc6caUiabc6caUiabdchaWnaaBaaaleaacqWGXbqCdaWgaaadbaGaemOta4eabeaaaSqabaGccqGGSaalaaa@5DE8@

where the sum is over all distinct combinations of the integers *q*_*i *_(the counts resulting from each of the *N *branching processes) that satisfy ∑i=0Nqi=M
 MathType@MTEF@5@5@+=feaafiart1ev1aaatCvAUfKttLearuWrP9MDH5MBPbIqV92AaeXatLxBI9gBaebbnrfifHhDYfgasaacH8akY=wiFfYdH8Gipec8Eeeu0xXdbba9frFj0=OqFfea0dXdd9vqai=hGuQ8kuc9pgc9s8qqaq=dirpe0xb9q8qiLsFr0=vr0=vr0dc8meaabaqaciaacaGaaeqabaqabeGadaaakeaadaaeWaqaaiabdghaXnaaBaaaleaacqWGPbqAaeqaaOGaeyypa0Jaemyta0ealeaacqWGPbqAcqGH9aqpcqaIWaamaeaacqWGobGta0GaeyyeIuoaaaa@3847@. It is clear that computing this quantity rapidly becomes impractical as the number of cells or the number of divisions increases, even in this simple single-type example. To use it with the multi-type branching processes we deal with here is essentially impossible; hence the moment-based approach we take in this paper.

**Table 1:** Parameter estimates with synthetic data.

**Model 1**				
			Proportion of simulations within	
Par	True	Mean (SD)	95% CI	99% CI

*γ*_0_	0.2	0.200 (0.003)	0.947	0.989
*δ*_0_	0.7	0.700 (0.002)	0.951	0.990
*γ*_1_	0.7	0.700 (0.006)	0.949	0.990
*δ*_1_	0.25	0.250 (0.007)	0.950	0.991

**Model 2**				
			Proportion of simulations within	

Par	True	Mean (SD)	95% CI	99% CI

*γ*_0_	0.2	0.200 (0.002)	0.950	0.989
*δ*_0_	0.7	0.700 (0.003)	0.952	0.990
*γ*_1_	0.7	0.700 (0.005)	0.951	0.989
*δ*_1_	0.25	0.250 (0.004)	0.950	0.990

**Model 3**				
			Proportion of simulations within	

Par	True	Mean (SD)	95% CI	99% CI

*f*_*A*_	0.1	0.101 (0.013)	0.953	0.987
γ0A MathType@MTEF@5@5@+=feaafiart1ev1aaatCvAUfKttLearuWrP9MDH5MBPbIqV92AaeXatLxBI9gBaebbnrfifHhDYfgasaacH8akY=wiFfYdH8Gipec8Eeeu0xXdbba9frFj0=OqFfea0dXdd9vqai=hGuQ8kuc9pgc9s8qqaq=dirpe0xb9q8qiLsFr0=vr0=vr0dc8meaabaqaciaacaGaaeqabaqabeGadaaakeaaiiGacqWFZoWzdaqhaaWcbaGaeGimaadabaGaemyqaeeaaaaa@3080@	0.15	0.150 (0.041)	0.943	0.984
δ0A MathType@MTEF@5@5@+=feaafiart1ev1aaatCvAUfKttLearuWrP9MDH5MBPbIqV92AaeXatLxBI9gBaebbnrfifHhDYfgasaacH8akY=wiFfYdH8Gipec8Eeeu0xXdbba9frFj0=OqFfea0dXdd9vqai=hGuQ8kuc9pgc9s8qqaq=dirpe0xb9q8qiLsFr0=vr0=vr0dc8meaabaqaciaacaGaaeqabaqabeGadaaakeaaiiGacqWF0oazdaqhaaWcbaGaeGimaadabaGaemyqaeeaaaaa@307E@	0.70	0.706 (0.055)	0.951	0.981
γ1A MathType@MTEF@5@5@+=feaafiart1ev1aaatCvAUfKttLearuWrP9MDH5MBPbIqV92AaeXatLxBI9gBaebbnrfifHhDYfgasaacH8akY=wiFfYdH8Gipec8Eeeu0xXdbba9frFj0=OqFfea0dXdd9vqai=hGuQ8kuc9pgc9s8qqaq=dirpe0xb9q8qiLsFr0=vr0=vr0dc8meaabaqaciaacaGaaeqabaqabeGadaaakeaaiiGacqWFZoWzdaqhaaWcbaGaeGymaedabaGaemyqaeeaaaaa@3082@	0.70	0.699 (0.020)	0.951	0.990
δ1A MathType@MTEF@5@5@+=feaafiart1ev1aaatCvAUfKttLearuWrP9MDH5MBPbIqV92AaeXatLxBI9gBaebbnrfifHhDYfgasaacH8akY=wiFfYdH8Gipec8Eeeu0xXdbba9frFj0=OqFfea0dXdd9vqai=hGuQ8kuc9pgc9s8qqaq=dirpe0xb9q8qiLsFr0=vr0=vr0dc8meaabaqaciaacaGaaeqabaqabeGadaaakeaaiiGacqWF0oazdaqhaaWcbaGaeGymaedabaGaemyqaeeaaaaa@3080@	0.20	0.200 (0.035)	0.942	0.981
*γ*^*B*^	0.40	0.400 (0.003)	0.955	0.992
*δ*^*B*^	0.35	0.350 (0.003)	0.946	0.983

For each of the models illustrated in Figure 2 we show the true parameter values and the mean and standard deviation of the QL parameter estimates generated from 10000 simulated datasets.  As a check of the validity of the assumption of normality of the QL estimators, we indicate the proportion of the simulations in which the true parameters lay within the predicted 95 and 99% confidence intervals obtained from the information matrix evaluated at the QL estimate

**Table 2:** Parameter estimates for the best fit description of the T cell proliferation data.

		95% confidence intervals		
Parameter	QL estimate	From QL alone	Monte Carlo with EM	Monte Carlo with EM + 5% noise

*γ*_0_	**0.221**	(0.211, 0.230)	(0.203, 0.339)	(0.181, 0.345)
*α*_0_	**0.232**	(0.222, 0.242)	(0.175, 0.253)	(0.163, 0.283)
*γ*_1–3_	**0.419**	(0.409, 0.430)	(0.365, 0.443)	(0.356, 0.444)
*α*_1–3_	**0.427**	(0.412, 0.442)	(0.379, 0.582)	(0.348, 0.595)
*γ*_4_	**0.086**	(0.077, 0.096)	(0.067, 0.679)	(0.063, 0.478)
*α*_4_	**0.340**	(0.244, 0.437)	(0.027, 0.691)	(0.094, 0.731)

Subscripts on the QL estimates of parameters refer to generation numbers – e.g., _*γ*1–3_ is the division probability in each 12 h timestep for cells in generations 1–3. We show the 95% confidence intervals obtained (i) using the asymptotic normality of the QL estimator, assuming no uncertainty in cell counts, (ii) by generating Monte Carlo (MC) samples from the original CFSE profiles using the EM method as described in the text, and (iii) using the MC method but also adding 5% noise to total counts as an estimate of error in the estimation of total cell counts.
